# Design and Development of Xanthone Hybrid for Potent Anti‐Inflammatory Effects: Synthesis and Evaluation

**DOI:** 10.1111/jcmm.70477

**Published:** 2025-03-24

**Authors:** Shreyasi Karmakar, Riya Saikia, Aparoop Das, Kalyani Pathak, Padmashree Das, Biman Bhuyan, Taha Alqahtani, Humood Al Shmrany, Bikram Dhara, Ajoy Kumer

**Affiliations:** ^1^ Department of Pharmaceutical Sciences Dibrugarh University Dibrugarh Assam India; ^2^ Centre for Biotechnology and Bioinformatics Dibrugarh University Dibrugarh Assam India; ^3^ Department of Pharmacology, College of Pharmacy King Khalid University Abha Saudi Arabia; ^4^ Department of Medical Laboratory Sciences, College of Applied Medical Sciences Prince Sattam Bin Abdulaziz University Al‐Kharj Saudi Arabia; ^5^ Center for Global Health Research, Saveetha Medical College and Hospital Saveetha Institute of Medical and Technical Sciences Chennai India; ^6^ Department of Chemistry, College of Arts and Sciences IUBAT‐International University of Business Agriculture and Technology, 4 Embankment Drive Road, Sector 10, Uttara Model Town Dhaka‐1230 Bangladesh

**Keywords:** COX‐2 enzyme, cytokines, hybrid‐xanthone, in silico study, in vitro study, in vivo study, inflammation

## Abstract

Inflammatory responses, while essential for host defence, can precipitate chronic pathologies when sustained. The polyphenolic entity xanthone is distinguished by its capacity to modulate inflammation, notably via the inhibition of the COX‐2 enzyme and associated inflammatory pathways. Additionally, heterocyclic frameworks such as pyrazole, triazole, and imidazole are recognised for their anti‐inflammatory attributes. This investigation was conducted to engineer and synthesise a series of novel hybrid‐xanthone molecules with enhanced anti‐inflammatory capabilities. Utilising computational docking strategies, these hybrid‐xanthone variants were virtually screened against the COX‐2 enzyme structure (PDB ID:1CX2), and the 10 leading candidates were identified based on their binding affinities. These selected entities were synthesised through an optimised three‐stage synthetic route. Subsequent in vitro assessments were performed using the Egg albumin denaturation assay at incremental concentrations. Complementary in vivo experiments involved the Carrageenan‐induced paw edema protocol in Wistar rats, administered at 200 mg/kg to evaluate the anti‐inflammatory response over a period of 6 h. The best percentage inhibition was shown by compound A127**(3‐(5′(1,2,4‐Triazole)‐pentyloxy)‐1,6,8‐trihydroxy xanthone)**, A11**(3‐(1′‐(1,2,4‐Triazole)‐methyloxy)‐1,6,8‐trihydroxy xanthone)** and A119**(3‐(1′‐(1,2,4‐Triazole)‐methyloxy)‐1,6,8‐trihydroxy xanthone)** as 60 ± 0.31, 58.57 ± 0.023, and 57.14 ± 0.21 respectively. Spectroscopic characterisation of the compounds was achieved through UV, IR, NMR, and Mass spectrometry techniques. The investigation revealed that out of the synthesised cohort, nine compounds exhibited favourable *in silico* profiles, and half of these manifested substantial anti‐inflammatory efficacy in both in vitro and in vivo models, outperforming the reference standard. These hybrid‐xanthone molecules demonstrated precise COX‐2 inhibition and maintained an acceptable safety margin in vivo, underscoring their therapeutic promise as anti‐inflammatory agents.

## 1 Introduction

Inflammation serves as a sophisticated biological process integral to the onset and progression of myriad health conditions. This intricate mechanism acts as a protective shield, defending the body against microbial invasion and tissue damage. The ultimate goal of this response is to repair tissue damage, eradicate harmful agents, and restore homeostasis. However, when inflammation becomes excessive, it can pave the way to severe pathological conditions [1]. Chronic inflammation is implicated in a large spectrum of diseases, encompassing hay fever, rheumatoid arthritis, atherosclerosis, multiple sclerosis, and conditions such as obesity‐related diabetes, tuberculosis, vasculitis, asthma, syphilis, and celiac disease [2, 3]. The orchestration of the inflammatory response is a complex interplay involving macrophages and neutrophils, which are pivotal in initiating, sustaining, and ultimately resolving inflammation. The acute phase is marked by increased vascular permeability, augmented blood flow, and the accumulation of leukocytes, fluids, and inflammatory mediators. Both phases are influenced by factors that promote leukocyte recruitment through the upregulation of cell adhesion molecules and chemotactic factors [4, 5]. Chronic inflammation, often associated with conditions like cardiovascular disease and type 2 diabetes mellitus, emerges not necessarily from traditional inflammatory triggers such as injury or infection but from tissue dysfunction and homeostatic imbalances across various physiological systems unrelated to tissue repair or defence [6, 7].

Cyclooxygenases (COX) are pivotal enzymes in the inflammatory cascade, primarily involved in the biosynthesis of prostanoids, which are precursors to prostaglandins, thromboxanes, and prostacyclins—key mediators of inflammation [8]. COX enzymes, released from cell membranes by the action of phospholipase A2, are categorised into three isoforms: COX‐1, COX‐2, and COX‐3 [9]. COX‐1 is constitutively expressed, regulating normal physiological functions, while COX‐2 is inducible and responds to inflammatory stimuli such as growth factors and cytokines, leading to the production of pro‐inflammatory prostaglandins [10]. Anti‐inflammatory drugs, particularly nonsteroidal anti‐inflammatory drugs (NSAIDs), target the COX enzymes to control inflammation [11]. Although NSAIDs' therapeutic effects are attributed to COX‐2 inhibition, nonspecific inhibition of COX‐1 can result in adverse effects. This realisation prompted the development of selective COX‐2 inhibitors, which, despite reducing side effects, were later associated with cardiovascular risks, leading to their market withdrawal and a shift towards exploring new COX‐2 inhibitory scaffolds [12, 13].

Xanthones, a class of secondary metabolites, are celebrated for their structural diversity and a broad spectrum of biological activities, including anti‐diabetic, anti‐microbial, anti‐oxidant, anti‐cancer, and cardioprotective effects [14]. Predominantly found in the plant families Gentianaceae, Moraceae, Clusiaceae, and Polygalaceae, xanthones possess a distinctive C6‐C1‐C6 carbon skeleton, with two aromatic rings linked by an ether and a carbonyl group. Ring A is derived from the acetate pathway, while ring B originates from the shikimic acid pathway, culminating in the formation of the xanthone core, also known as 9H‐xanthen‐9‐one [15]. Xanthones have demonstrated significant anti‐inflammatory activity by showcasing inhibition of various enzymes (like LOX, CPX‐2) and pro‐inflammatory cytokines (like IL‐1β, TNF‐α, IL‐6) which are responsible for inflammation. Other than this, due to the anti‐oxidation property of xanthone, it can reduce oxidative stress related to inflammation, and xanthones are also involved in controlling signalling pathways associated with it, like MAPK and NF‐κB pathways [16]. Complementing xanthones, heterocyclic compounds such as pyrazole and 1,2,4‐triazole have also been reported to exhibit anti‐inflammatory properties [17]. This property of both the xanthones and heterocycles has led to the synthesis of xanthone‐hybrid molecules. The hybrid‐xanthone molecules are accepted to potentially enhance the therapeutic potential of the molecule in juxtaposition to either xanthone or heterocycles. Therefore, the study involves the design, synthesis, and evaluation of the anti‐inflammatory potential of hybrid xanthone derivatives, incorporating pyrazole and 1,2,4‐triazole heterocycles, to elucidate their therapeutic role in inflammation management.

## 2 Materials and Methods

### 2.1 Ligand Designing and In Silico Study

The ligands are designed and drawn into 2D cdx format, using Chem Draw Ultra 8.0 software, which is then transformed into 3D mol format by using the same. These ligands, with the use of Molinspiration software, physiochemical properties were studied. Subsequently, toxicity and ADME studies were carried out using ORISIS data warrior and SWISS ADME. After the collection of protein from the Protein Data Bank (http://www.pdb.org) in pdb text format followed by molecular docking was performed using Autodock 4.2 [18]. This study's purpose is to provide insight into the binding propensities of ligands having the highest affinity for the target protein as appeared from virtual screening. Autodock v 4.2.6 was used to perform docking investigations on these molecules in question. Both these receptor and target compounds were then saved in pdbqt format after combining non‐polar hydrogens. Molecular docking was performed within a grid box dimension of 18.12 × 19.84 × 15.95 Å for the protein. It was necessary to design grid boxes with particular dimensions and 0.3 Å spacing. Docking experiments of the protein‐ligand complex were carried out in accordance with the Lamarckian Genetic Algorithm (LGA). There were three replicates of molecular docking investigations, each of which included 50 solutions, a population size of 500, 2,500,000 evaluations, a maximum generational number of 27, and all other parameters left at their default values. Thereafter, the best 10‐docked compounds with minimum binding energy were utilised to carry out the synthesis and in vivo studies.

### 2.2 Molecular Dynamics Simulation (MDS) Study

MD simulation was executed on the optimal docked complex of the best ligand and target protein EGFR complex using Desmond 2020.1 [1]. The simulations utilised the OPLS‐2005 force field [2, 3, 4] and explicit solvent model with SPC water molecules [5] within a periodic boundary solvation box with dimensions of 1.0 Å × 1.0 Å × 1.0 Å. To neutralise the electrical charge, sodium ions (Na^+^) were added. A 0.15 M NaCl solution was introduced to mimic the physiological environment. The system was gradually equilibrated by simulating the protein‐ligand complexes under an NVT ensemble for 10 ns. Subsequently, an NPT ensemble was employed for a brief 12‐ns equilibration and minimisation run. The Nose‐Hoover chain coupling method [6] was utilised to maintain a constant temperature with a relaxation time of 1.0 ps and pressure of 1 bar. A time step of 2 femtoseconds was used in the simulations. Pressure regulation was achieved using the Martyna‐Tuckerman‐Klein chain coupling scheme barostat technique [7], with a relaxation period of 2 ps. Long‐range electrostatic interactions were computed using the particle mesh Ewald approach [8], with a coulomb interaction radius of 9 Å. The RESPA integrator was employed to calculate bonded forces with a time step of 2 fs. The final production run was performed for a duration of 100 ns per system. To assess the stability of the MD simulations, key parameters were analysed, including root mean square deviation (RMSD), radius of gyration (Rg), root mean square fluctuation (RMSF), and number of hydrogen bonds (H‐bonds). These parameters provided insights into the conformational changes and stability of the protein‐ligand complexes throughout the simulations.

### 2.3 Chemistry

The chemicals used in the synthesis were of analytical grade; 2,4,6‐trihydroxybenzoic acid was purchased from Sigma‐Aldrich, USA, while the rest of the chemicals were from commercial distributors and were utilised without additional purification unless specified. Further, to mark the completion of synthesis, a thin layer chromatography (TLC) test was carried out, along with which characterisation and spotting were done. A melting point and solubility study was also done. Moreover, with the use of Fourier‐transform infrared spectroscopy (FT‐IR) (Nicolet, iS10 Thermo Scientific, Madison, USA), the spectrum was taken down. 1H and 13C NMR spectra were observed for the synthesised compound, followed by the mass spectrum with Waters Q‐mass spectrometer (Waters, Q‐ToFMICROMASS, United States).

### 2.4 General Scheme

**SCHEME 1:**
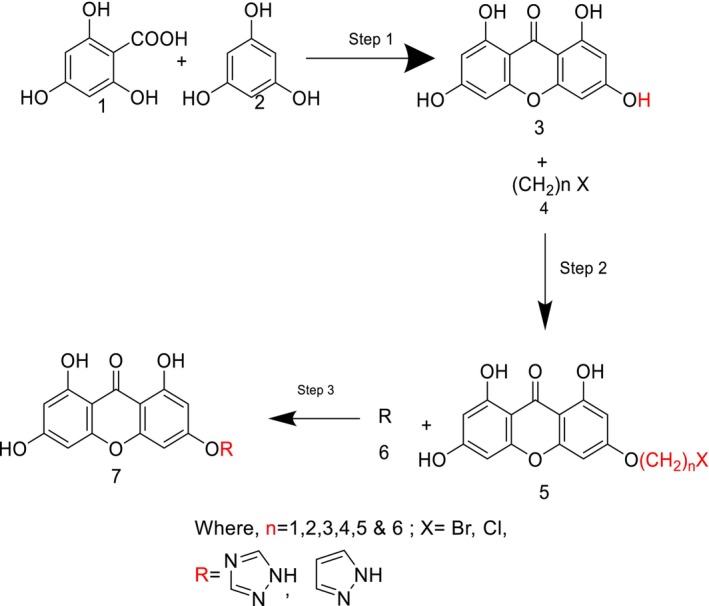
Pathway for the synthesis of the hybrid xanthone derivatives; Reagents and conditions; (Step 1) At a temperature of 70°C for 35 min Eaton's reagent is stirred, subsequently stirred in ice at 3°C–5°C for 2 h 35 min (Step 2) Acetone, K_2_CO_3_, alkyl halide (R‐Br, R‐I), refluxed for 8 h at 60°C (Step 3) Acetone, K_2_CO_3_, heterocyclic compound, refluxed at room temperature for 48 h.

The synthesis of the xanthone moiety was initiated by reacting 2,4,6‐trihydroxybenzoic acid and phloroglucinol in the presence of Eaton's reagent (phosphorus pentoxide dissolved in methane sulphonic acid in a 1:10 ratio) at 70°C. The mixture is then cooled, poured into ice, and held there at 0°C–4°C for 2 h and 30 min (Scheme 1). Then, filtration of the collected residue was carried out, and it was rinsed with water until pH 6 was obtained (Step 1). The process is then followed by alkylation of the xanthone moiety, where the solids, after drying, are treated with acetone (50–60 mL), potassium carbonate (2.5 mmol) and haloalkane (3 mmol) to be refluxed for 8 h at 60°C under stirring. The reacted mixture was filtered, and a pale yellow solid was collected (intermediate 2) (Step 2). The pale yellow solid was then treated with a selected heterocyclic compound (20 mmol) to give the hybrid xanthone derivative in the presence of potassium carbonate (3 mmol) and acetone (20 mL). This mixture was refluxed for 48 h at room temperature (Step 3) and was finally cooled, filtered, recrystallized, and brown solids were collected [19, 20].

### 2.5 Physicochemical Evaluation of the Synthesised Compounds

The characterisation and identification of the synthesised compounds were achieved by Thin layer chromatography (TLC) test, including the completion of the reaction. With the use of Silica gel G, TLC plates were prepared, and the mobile phase consisted of acetone and hexane (ratio 2:1) [21]. The melting point equipment was used to check the melting point. A solubility study was carried out using different solvents of extended polarities. Each chemical (0.2 mg) was introduced to the solvent of 2 mL [22]. Finally, by the use of a UV–VIS spectrophotometer, the synthesised products are characterised to note the λ_max_, where 0.1 mg of each product is dissolved in 10 mL of solvent, with additional dilution to measure the lmax. The maximum absorption wavelength (lmax) is noted on the UV–VIS spectrophotometer instrument. After this, FT‐IR spectrums are recorded, and by the use of water as solvent, 1H and 13C NMR at 100 MHz are recorded respectively. Further, the mass spectrum data are obtained by the peaks of the m/z ratio in the obtained spectra [23, 24].

### 2.6 Pharmacological Evaluation

#### 2.6.1 In Vitro Study‐Egg Albumin Denaturation Method

For the synthesised product, in vitro protein denaturation was carried out. In this, the reaction mixture (5 mL) comprises 0.2 mL egg albumin (from fresh hen's egg), 2.8 mL of phosphate‐buffered saline (pH 6.4), and 2 mL of different concentrations (100, 250, 500 μg/mL) of the drug. Likewise, distilled water served as a control. Now, the mixture was incubated for 15 min at 37°C ± 2°C and heated for 5 min at 70°C. After cooling, the absorbance was assessed at 660 nm using the vehicle as a blank. Indomethacin was used as a reference at a similar concentration and was similarly treated for absorbance determination [25, 26]. The percentage inhibition of the protein denaturation was enumerated using the following formula:

$$\%\text{inhibition}=100\times \left[V_{t}/V_{c}-1\right]$$

where, V_*t*_ = absorbance of the test sample, V_*c*_ = absorbance of control.

#### 2.6.2 In Vivo Study: Evaluation of Anti‐Inflammatory Activity

##### 2.6.2.1 Selection of Animals

Female adult Wistar rats of weight around 100–150 g were utilised for the study. All studies were conducted under the guidelines promulgated by the Institutional Animal Care Committee, CPCSEA, India (approval no. IAEC/DU/244, dated. 17 December 2022). All studies, particularly those that include animals, were conducted under stern adherence to the guidelines and ethics for the tests involving the animal subjects.

##### 2.6.2.2 Maintenance and Adjustment of the Selected Animals

All animals used in the experiment were kept in spacious animal houses having proper ventilation and well‐labelled cages. The room temperature was maintained at 24°C ± 2°C, at RH of 44%–56%, and a normal display of a 12 h light/dark cycle during the study, as per the standard requirements stated in the OECD guidelines. They were also given unlimited access to water *ad libitum* and rodent pellets.

### 2.7 Acute Toxicity Study

As specified in OECD guideline‐425, an acute toxicity study was conducted for the 10 synthesised hybrid xanthone derivatives using female albino mice. All 10 compounds were taken at dose levels of 175, 550, and 2000 mg/kg body weight, dissolved in 0.5% Carboxymethyl cellulose (CMC). The effective dose was computed as 1/10 of the maximal dose.

### 2.8 Chemicals and Reagents

Carboxymethyl cellulose (CMC), Carrageenan, Celecoxib (standard drug) and all the other chemicals used for the animal experiments are of analytical grade.

### 2.9 Evaluation of Anti‐Inflammatory Activity

#### 2.9.1 Carrageenan Induced Paw‐Edema Method

For carrying out the animal experiment for anti‐inflammatory activity, animals were arranged in a group of 12 (*n* = 5). In the hind paw of each rat, the freshly prepared Carrageenan (1.0% w/v, 0.1 mL) suspension was injected. Here the negative control (Group I) is served with only CMC as the vehicle. The standard Celecoxib 10 mg/kg body weight in 0.5% CMC was received by Group II, while the rest of Groups III–XII were treated with the test samples (200 mg/kg body weight administered orally). After the completion of 1 h, all the rats were injected with a freshly prepared solution of Carrageenan (1.0% w/v) in their right hind paws [27, 28, 29]. By the use of a plethysmometer, the swelling volume of the right hind paw was estimated at times 0, 1, 2, 3, 4 and 6 h. For the determination of percentage inhibition, the following formula was used:

$$\%\text{inhibition}=100\times \left[V_{t}/V_{c}-1\right]$$

where, V_*t*_ = indicates the mean increase in paw volume of the treated group. V_*c*_ = indicates the mean increase in paw volume of the control group.

#### 2.9.2 Serum Inflammatory Marker Assessment

The serum levels of anti‐inflammatory and pro‐inflammatory IL‐4, IL‐10, IL‐6, and TNF‐α were measured by the use of commercial sandwich ELISA (Enzyme‐Linked Immunosorbent Assay) kits as per the instruction of the manufacturer. For this, three rats were used in the case of each group for cytokine analysis at the end of the treatment. The blood was collected from the retro‐orbital plexus of the rats into heparinized tubes. After this, serum was isolated by centrifugation at 1500 rpm for 15 to 20 min and then frozen at −20°C until the ELISA study had been carried out [30].

### 2.10 Statistical Analysis

The data are analysed by one‐way ANOVA test and Dennett's test, and the results of the mean are expressed as SEM. The significance of the result is considered at *p* < 0.05. GraphPad Prism version 9.5.1(733) has been used to carry out all the statistical calculations. All the values are expressed as Mean ± SEM (*n* = 6), Standard vs. all group (**p < 0.01, *p < 0.05).

### 2.11 QSAR Model Building for Physicochemical Attributes Determination of XOS

#### 2.11.1 Collection of Dataset

For this study, we chose 67 molecules as inhibitors for COX‐2 and COX‐1 proteins from the chEMBL database (https://www.ebi.ac.uk/chembl/document_report_card/CHEMBL1142830/) for anti‐inflammation effectiveness (experimental IC50 = 149 nM to 7 mM). pIC50 was calculated from the IC50 results (pIC50 = −logIC50) as a training set in preparation for the QSAR study. In this study, three of the least active and five of the most active molecules are shown to illustrate the range of bioactivity with chemical characteristics covered by the current dataset. For all the compounds, the reported IC50 and pIC50 values are presented as SMILES strings.

#### 2.11.2 Preparation of Structures and Energy Minimization

The structures of individual inhibitors were fetched from the chEMBL public domain database (https://www.ebi.ac.uk/chembl). All the SMILE strings were subjected to structure building, using OpenBabel 2.4.0, another free and open‐source chemical toolkit, to transform these 2D structures into their 3D counterparts. The molecule was then optimised using TINKER's built‐in force field MMFF94 and aligned with Open3DAlign's default parameters.

#### 2.11.3 Calculations of Molecular Descriptors

Padel descriptor calculated various molecular descriptors for each molecule. More than 15,000 molecular descriptors had been provided by PyDescriptor for each molecule. It is necessarily important to remove redundant molecular descriptors to steer clear of the impinging of multi‐collinearity and spurious variables in the GA‐MLR model. Hence, molecular descriptors with nearly constant values (> 95%) and co‐linearity (|*R*|) > 0.95 were removed using objective feature selection (OFS) in QSARINS v2.2.4 [31, 32, 33]. The contracted molecular descriptor pool thus resulted is comprised of 1370 molecular descriptors only.

#### 2.11.4 Construction and Verification of a Quantitative Structure–Activity Relationship Model

The reduced molecular descriptor collection was rich in diversity, including 0D to 3D descriptors, charge descriptors, molecular characteristics, and more. To create statistically sound QSAR models using GA‐MLR, QSARINS v2.2.4 used subjective feature selection (SFS). After this, the generated models were subjected to a stringent round of statistical validation (both internally and externally), Y‐randomization, and application domain analysis in accordance with OECD guidelines. When used as a training dataset, 45 molecules are sufficiently big to cover a wide range of chemical space. The methodology for building a QSAR model from a subset of the dataset is as follows:

An internal and external validation of the created QSAR model(s), for instance, is required by OECD recommendations. So, some molecules were selected at random from the dataset and used as a prediction set, while the other molecules were used as a training set, and SFS was applied to them in order to create the QSAR model. Molecules from the prediction set are used to test the accuracy of the QSAR model(s) created.

Using the random splitting option in QSARINS v.2.2.4, the dataset was randomly divided into a training set consisting of 45 molecules (the training set) and a prediction set consisting of five molecules (the prediction set). QSAR models were developed using the training set, whereas the prediction set was used for independent verification. For the birth of the GA‐MLR‐based QSAR models, we used QSARINS v2.2.4 with the default parameters and Q2LOO as the fitness function for feature selection using double cross validation. The Q2LOO value was shown to grow dramatically up to six variables, and then just a little beyond that. To avoid the risk of overfitting, we only included a small subset of molecular descriptors (there are six total), which allowed us to easily and accurately generate QSAR models (see Table S3 for values of all molecular descriptors used in QSAR models). The models were validated internally and externally, Y‐randomised, and subjected to an examination of their application domain (ad) using QSARINS, as recommended by the OECD, to guarantee their accuracy.

Using the following criteria: (a) internal validation using the Leave‐One‐Out (LOO) and Leave‐Many‐Out (LMO) procedures; (b) external validation; (c) Y‐randomization; and (d) meeting the corresponding threshold value for the statistical parameters: *R* ^2^ 0.6, *Q* ^2^LOO 0.5, *Q* ^2^LMO 0.6, *R* ^2^ > *Q* ^2^, *R* ^2^ex 0.6, RMSEtr RMSE. None of the QSAR models that did not fit these requirements were included. For this reason, the two QSAR models (1.1 and 1.2) with the best values of these parameters and the highest predictive abilities (*Q* ^2^−Fn > 0.71) were chosen.

## 3 Result and Discussion

### 3.1 Drug Likeness Study

The designed xanthone molecules with different substitutions on the xanthone moiety were subjected to pharmacokinetic and toxicological studies. The HBD (hydrogen bond donor), volumes, HBA (hydrogen bond acceptor), TPSA (total polar surface area), nRB (number of rotatable bonds), molecular weight, and volumes were calculated by the use of Molinspiration's property calculator, as shown in Table 1. Tables 2 and 3 consist of the toxicity profile and ADME study, which were predicted using ORISIS data Warrior and SWISS ADME, respectively.

**TABLE 1:** Analysis of the various molecular properties of the designed compounds (the synthesised compounds).

Compouds code	Molecular weight of compounds	Molecular formula of compounds	MlogP values	nRB	TPSA	nOH	nOH‐NH	nViolations	Volume
A1	410.43		4.14	8	117.95	8	3	0	359.05
A2	546.62		5.80	15	124.79	10	2	2	496.64
A3	340.29		2.58	3	117.95	8	3	0	275.04
A4	420.38		3.18	6	124.79	10	2	0	345.43
A5	354.32		2.59	4	117.95	8	3	0	291.84
A6	448.44		3.20	8	124.79	10	2	0	379.03
A7	368.35		2.86	5	117.95	8	3	0	308.64
A8	476.49		3.74	10	124.79	10	2	0	412.63
A9	382.37		3.13	6	117.95	8	3	0	325.44
A10	504.54		4.28	12	124.79	10	2	1	446.24
A11	396.40		3.64	7	117.95	8	3	0	342.25
A12	532.60		5.29	14	124.79	10	2	2	479.84
A13	461.51		5.34	8	101.16	7	3	1	413.43
A14	644.77		8.91	15	99.00	8	2	2	592.94
A15	389.36		4.42	3	105.06	7	3	0	323.19
A16	518.52		6.86	6	99.00	8	2	2	441.72
A17	403.39		4.43	4	105.06	7	3	0	339.99
A18	546.58		6.88	8	99.00	8	2	2	475.33
A19	417.42		4.70	5	105.06	7	3	0	356.79
A20	574.63		7.42	10	99.00	8	2	2	508.93
A21	432.44		4.97	6	105.06	7	3	0	373.59
A22	602.69		7.97	12	99.00	8	2	2	542.53
A23	445.47		5.48	7	105.06	7	3	1	390.39
A24	630.74		8.68	14	99.00	8	2	2	576.14
A25	475.45		4.15	7	139.21	9	3	0	400.94
A26	690.71		6.32	14	167.29	12	2	3	597.24
A27	662.65		5.31	12	167.29	12	2	3	563.63
A28	461.43		3.64	6	139.21	9	3	0	384.14
A29	447.40		3.37	5	139.21	9	3	0	367.34
A30	634.60		4.76	10	167.29	12	2	2	530.03
A31	606.54		4.22	8	167.29	12	2	2	496.43
A32	433.37		3.10	4	139.21	9	3	0	350.54
A33	419.35		3.09	3	139.21	9	3	0	333.74
A34	578.49		4.20	6	167.29	12	2	2	462.82
A35	704.73		6.82	15	167.29	12	2	3	614.04
A36	491.50		4.01	8	135.30	9	3	0	423.98
A37	410.43		3.75	8	117.95	8	3	0	359.05
A38	560.65		5.52	16	124.79	10	2	2	496.62
A39	420.38		2.39	6	124.79	10	2	0	345.43
A40	340.29		2.19	3	117.95	8	3	0	275.04
A41	354.32		2.20	4	117.95	8	3	0	291.84
A42	448.44		2.42	8	124.79	10	2	0	379.03
A43	476.49		2.96	10	124.79	10	2	0	412.63
A44	368.35		2.47	5	117.95	8	3	0	308.64
A45	382.37		2.74	6	117.95	8	3	0	325.44
A46	504.54		3.50	12	124.79	10	2	1	446.24
A47	532.60		4.51	14	124.79	10	2	1	479.84
A48	396.40		3.25	7	117.95	8	3	0	342.25
A49	428.49		3.40	8	115.39	8	4	0	387.98
A50	582.74		4.30	15	119.67	10	4	1	554.51
A51	456.50		1.68	6	119.67	10	4	0	403.29
A52	358.35		1.83	3	115.39	8	4	0	303.97
A53	327.38		1.84	4	115.39	8	4	0	320.77
A54	484.55		1.71	8	119.67	10	4	0	436.89
A55	386.40		2.11	5	115.39	8	4	0	337.57
A56	512.61		2.25	10	119.67	10	4	1	470.50
A57	400.43		2.38	6	115.39	8	4	0	354.38
A58	540.66		2.79	12	119.67	10	4	1	504.10
A59	414.46		2.89	7	115.39	8	4	0	371.18
A60	568.72		3.80	14	119.67	10	4	1	537.71
A61	341.28		1.65	3	130.85	9	3	0	270.88
A62	422.36		1.32	6	150.57	12	2	1	337.11
A63	355.31		1.66	4	130.85	9	3	0	287.68
A64	450.41		1.35	8	150.57	12	2	1	370.72
A65	369.33		1.94	5	130.85	9	3	0	304.49
A66	478.46		1.89	10	150.57	12	2	1	404.32
A67	383.36		2.21	6	130.85	9	3	0	321.29
A68	506.52		2.43	12	150.57	12	2	2	437.92
A69	397.39		2.71	7	130.85	9	3	0	338.09
A70	534.57		3.44	14	150.57	12	2	2	471.53
A71	411.41		3.22	8	130.85	9	3	0	354.89
A72	562.63		4.45	16	150.57	12	2	2	505.13
B1	394.43		4.67	8	97.73	7	2	0	351.03
B2	324.29		3.11	3	97.73	7	2	0	267.02
B3	338.32		3.12	4	97.73	7	2	0	283.82
B4	352.35		3.39	5	97.73	7	2	0	300.62
B5	366.37		3.66	6	97.73	7	2	0	317.43
B6	380.40		4.17	7	97.73	7	2	0	334.23
B7	443.50		6.51	8	84.83	6	2	1	399.18
B8	373.36		4.95	3	84.83	6	2	0	315.17
B9	387.39		4.96	4	84.83	6	2	0	331.97
B10	401.42		5.32	5	84.83	6	2	1	348.77
B11	415.44		5.50	6	84.83	6	2	1	365.57
B12	429.47		6.01	7	84.83	6	2	1	382.38
B13	473.48		5.18	8	118.98	8	2	1	409.73
B14	403.35		3.62	3	118.98	8	2	0	325.72
B15	417.37		3.63	4	118.98	8	2	0	342.52
B16	431.40		3.9	5	118.98	8	2	0	359.32
B17	445.43		4.17	6	118.98	8	2	0	376.12
B18	459.45		4.68	7	118.98	8	2	0	392.93
B19	394.43		4.28	8	97.73	7	2	0	351.03
B20	324.29		2.71	3	97.73	7	2	0	267.02
B21	352.35		3.00	5	97.73	7	2	0	300.62
B22	366.37		3.27	6	97.73	7	2	0	317.43
B23	380.40		3.77	2	97.73	7	2	0	334.23
B24	412.49		3.92	8	95.17	7	3	0	379.96
B25	342.35		2.36	3	95.17	7	3	0	295.95
B26	356.38		2.37	4	95.17	7	3	0	312.75
B27	384.43		2.91	6	95.17	7	3	0	346.36
B28	370.40		2.64	5	95.17	7	3	0	329.56
B29	398.46		3.42	7	95.17	7	3	0	363.16
C1	410.43		4.17	8	117.95	8	3	0	359.05
C2	340.29		2.61	3	117.95	8	3	0	275.04
C3	354.32		2.62	4	117.95	8	3	0	291.84
C4	382.37		3.16	6	117.95	8	3	0	325.44
C5	396.40		3.67	7	117.95	8	3	0	342.25
C6	445.47		5.51	7	105.06	7	3	1	390.39
C7	431.44		5.00	6	105.06	7	3	1	373.59
C8	417		4.73	5	105.06	7	3	0	356.79
C9	403.39		4.46	4	105.06	7	3	0	339.99
C10	389.36		4.45	3	105.06	7	3	0	323.19
C11	459.50		6.01	8	105.06	7	3	1	407.19
C12	489.48		4.68	8	139.21	9	3	0	417.75
C13	419.35		3.12	3	139.21	9	3	0	333.74
C14	433.37		3.13	4	139.21	9	3	0	350.54
C15	447.40		3.40	5	139.21	9	3	0	367.34
C16	475.45		4.18	7	139.21	9	3	0	400.94
C17	340.29		2.22	3	117.95	8	3	0	275.04
C18	354.32		2.23	4	117.95	8	3	0	291.84
C19	368.35		2.50	5	117.95	8	3	0	308.64
C20	382.37		2.77	6	117.95	8	3	0	325.44
C21	396.40		3.28	7	117.95	8	3	0	342.25
C22	428.49		3.43	8	115.39	8	4	0	387.98
C23	358.35		1.86	3	115.39	8	4	0	303.97
C24	372.38		1.87	4	115.39	8	4	0	320.77
C25	386.40		2.15	5	115.39	8	4	0	337.57
C26	400.43		2.42	6	115.39	8	4	0	354.38
C27	414.46		2.92	7	115.39	8	4	0	371.18

Abbreviations: nOH&NH, number of OH & NH bonds; nRB, number of rotatable bond; TPSA, total polar surface area. The colours are provided in order to demark the violated values.

**TABLE 2:** Toxicity data analysis of the designed compound by ORISIS DATA WARRIOR.

Compound no.	Mutagenic	Tumorigenic	Irritant	Reproductive effect
A1	High	None	Low	None
A3	High	None	Low	None
A4	High	None	High	None
A5	High	None	Low	None
A6	High	None	High	None
A7	High	None	Low	None
A8	High	None	High	None
A9	High	None	Low	None
A10	High	None	High	None
A11	High	None	Low	None
A13	High	None	Low	None
A15	High	None	Low	None
A16	High	None	High	None
A17	High	None	Low	None
A19	High	None	Low	None
A21	High	None	Low	None
A23	High	None	Low	None
A25	High	None	Low	None
A28	High	None	Low	None
A29	High	None	Low	None
A32	High	None	Low	None
A33	High	High	Low	High
A36	High	None	Low	None
A37	High	None	Low	None
A39	High	None	High	None
A40	High	None	Low	None
A41	High	None	Low	None
A42	High	None	High	None
A43	High	None	High	None
A44	High	None	Low	None
A45	High	None	Low	None
A46	High	None	High	None
A47	High	None	High	None
A48	High	None	Low	None
A49	High	None	Low	Low
A50	High	None	High	Low
A51	High	None	High	None
A52	High	None	Low	None
A53	High	None	Low	Low
A54	High	None	High	Low
A55	High	None	Low	Low
A56	High	None	High	Low
A57	High	None	Low	Low
A58	High	None	High	Low
A59	High	None	Low	Low
A60	High	None	High	Low
A61	High	None	Low	None
A62	High	None	High	None
A63	High	None	Low	None
A64	High	None	High	None
A65	High	None	Low	None
A66	High	None	High	None
A67	High	None	Low	None
A69	High	None	Low	None
A71	High	None	Low	None
B1	High	None	Low	None
B2	High	None	Low	None
B3	High	None	Low	None
B4	High	None	Low	None
B5	High	None	Low	None
B6	High	None	Low	None
B7	High	None	Low	None
B8	High	None	Low	None
B9	High	None	Low	None
B10	High	None	Low	None
B11	High	None	Low	None
B12	High	None	Low	None
B13	High	None	Low	None
B14	High	High	Low	High
B15	High	None	Low	None
B16	High	None	Low	None
B17	High	None	Low	None
B18	High	None	Low	None
B19	High	None	Low	None
B20	High	None	Low	None
B21	High	None	Low	None
B22	High	None	Low	None
B23	High	None	Low	None
B24	High	None	Low	None
B25	High	None	Low	Low
B26	High	None	Low	Low
B27	High	None	Low	Low
B28	High	None	Low	Low
B29	High	None	Low	Low
C1	High	None	Low	None
C2	High	None	Low	None
C3	High	None	Low	None
C4	High	None	Low	None
C5	High	None	Low	None
C6	High	None	Low	None
C7	High	None	Low	None
C8	High	None	Low	None
C9	High	None	Low	None
C10	High	None	Low	None
C11	High	None	Low	None
C12	High	None	Low	None
C13	High	None	Low	None
C14	High	High	Low	High
C15	High	None	Low	None
C16	High	None	Low	None
C17	High	None	Low	None
C18	High	None	Low	None
C19	High	None	Low	None
C20	High	None	Low	None
C21	High	None	Low	None
C22	High	None	Low	None
C23	High	None	Low	Low
C24	High	None	Low	None
C25	High	None	Low	Low
C26	High	None	Low	Low
C27	High	None	Low	Low

**TABLE 3:** Navigating the ADME properties of the designed compounds by SWISS ADME.

Compound no.	Log *p*	Solubility	Log kp (skin permeation)	Bioavailability score	Synthetic accessibility	GI absorption	BBB permeant
A1	3.59	−5.96	−6.11	0.55	3.39	High	No
A3	2.29	−4.58	−6.63	0.55	3.11	High	No
A4	2.9	−5.1	−6.86	0.55	3.47	High	No
A5	2.17	−4.43	−6.43	0.55	3.04	High	No
A6	3.39	−4.9	−7.17	0.55	3.55	High	No
A7	2.17	−4.85	−6.62	0.55	3.12	High	No
A8	3.71	−5.64	−6.84	0.55	3.72	High	No
A9	3.01	−5.21	−6.45	0.55	3.21	High	No
A10	3.96	−6.38	−6.5	0.55	3.94	Low	No
A11	3.26	−5.59	−6.28	0.55	3.29	High	No
A13	4.26	−7.53	−5.16	0.55	3.55	Low	No
A15	3.13	−6.14	−5.68	0.55	3.15	High	No
A16	4.56	−8.61	−4.61	0.55	4.57	Low	No
A17	3.49	−6.41	−5.67	0.55	3.23	High	No
A19	3.71	−6.78	−5.5	0.55	3.33	High	No
A21	3.9	−7.15	−5.33	0.55	3.43	High	No
A23	3.39	−6.76	−6.25	0.55	3.38	Low	No
A25	3.1	−6.39	−6.41	0.55	3.29	Low	No
A28	2.89	−6.02	−6.58	0.55	3.18	Low	No
A29	2.89	−6.02	−6.58	0.55	3.18	Low	No
A32	2.66	−5.65	−6.75	0.55	3.12	High	No
A33	1.92	−5.25	−6.55	0.55	3.26	High	No
A36	3.65	−7.13	−6.08	0.55	3.5	Low	No
A37	3.47	−5.73	−6.27	0.55	3.36	High	No
A39	3.08	−5.19	−6.63	0.55	3.58	High	No
A40	2.72	−4.64	−7.17	0.55	3.38	High	No
A41	3.72	−5.7	−6.05	0.55	3.28	High	No
A42	3.97	−5.6	−6.2	0.55	3.33	High	No
A43	4.78	−5.79	−6.74	0.55	3.83	High	No
A44	4.94	−6.16	−6.58	0.55	3.92	Low	No
A45	4.15	−5.97	−6.03	0.55	3.34	High	No
A46	4.43	−6.33	−5.78	0.55	3.5	High	No
A47	5.19	−7.28	−6.07	0.55	4.19	Low	No
A48	5.19	−7.28	−5.7	0.55	4.19	Low	No
A49	4.74	−6.72	−6.1	0.55	3.56	High	No
A50	5.43	−6.68	−7.14	0.55	3.92	High	No
A51	7.8	−7.95	−6.4	0.55	5.24	High	No
A52	5.44	−5.18	−6.55	0.55	4.39	High	No
A53	4.26	−5.3	−8.18	0.55	3.73	High	No
A54	4.55	−5.19	−7.12	0.55	3.64	High	No
A55	4.38	−3.63	−7.85	0.55	4.1	High	No
A56	3.24	−4.22	−6.21	0.55	3.31	High	No
A57	4.68	−4.37	−6.77	0.55	4.21	High	No
A58	4.91	−5.94	−6.05	0.55	3.37	High	No
A59	6.87	−4.95	−6.44	0.55	4.78	High	No
A60	5.06	−4.95	−5.77	0.55	3.81	High	No
A61	2.16	−4.61	−6.8	0.55	3.14	High	No
A62	2.28	−5.16	−7.2	0.55	3.46	Low	No
A63	2.09	−4.51	−6.96	0.55	3.13	High	No
A64	2.68	−4.96	−7.51	0.55	3.56	Low	No
A65	2.5	−4.88	−6.79	0.55	3.12	High	No
A66	3.21	−5.7	−7.17	0.55	3.68	Low	No
A67	2.78	−5.26	−6.61	0.55	3.21	High	No
A69	2.99	−5.62	−6.45	0.55	3.28	High	No
A71	3.54	−6.55	−5.98	0.55	3.51	Low	No
B1	3.81	−5.94	−6.28	0.55	4.99	High	No
B2	2.76	−6.46	−6.43	0.55	3.36	High	No
B3	2.17	−6.3	−5.53	0.55	3.07	High	No
B4	4.63	−7.2	−5.36	0.55	3.04	High	No
B5	4.86	−5.9	−5.19	0.55	3.38	High	No
B6	5.17	−4.52	−4.07	0.55	3.47	High	No
B7	6.07	−4.43	−4.59	0.55	3.55	High	No
B8	4.79	−6.14	−4.74	0.55	3.82	Low	No
B9	4.72	−6.51	−4.57	0.55	3.38	High	No
B10	5.19	−6.89	−4.4	0.55	3.44	High	No
B11	5.52	−8.81	−4.24	0.55	3.49	High	No
B12	5.72	−7.43	−4.98	0.55	3.59	High	No
B13	5.36	−7.33	−5.51	0.55	3.7	Low	No
B14	4.34	−7.7	−5.66	0.55	3.77	Low	No
B15	4.37	−8.08	−5.15	0.55	3.5	High	No
B16	4.37	−8.44	−5.15	0.55	3.38	High	No
B17	4.5	−8.43	−5.15	0.55	3.38	High	No
B18	4.5	−7.04	−5.7	0.55	3.65	High	No
B19	4.5	−6.94	−5.85	0.55	3.65	High	No
B20	4.16	−6.94	−5.69	0.55	3.65	High	No
B21	4.3	−8.05	−5.52	0.55	3.24	High	No
B22	4.51	−8.05	−5.35	0.55	3.3	High	No
B23	4.79	−8.05	−5.53	0.55	3.3	High	No
B24	4.97	−5.64	−6.05	0.55	3.41	High	No
B25	5.64	−5.55	−6.94	0.55	3.52	High	No
B26	4.61	−5.91	−6.61	0.55	3.88	High	No
B27	3.04	−6.28	−6.78	0.55	3.7	High	No
B28	3.86	−6.66	−6.44	0.55	3.3	High	No
B29	3.01	−6.61	−6.11	0.55	3.39	High	No
C1	4.1	−5.23	−6.63	0.55	3.28	High	No
C2	3.44	−3.79	−6.79	0.55	3.46	High	No
C3	2.13	−4.53	−6.62	0.55	3.48	High	No
C4	2.26	−4.15	−6.45	0.55	3.18	High	No
C5	2.77	−4.9	−6.28	0.55	3.16	High	No
C6	2.89	−5.96	−5.33	0.55	3.21	High	No
C7	3.21	−4.58	−5.5	0.55	3.3	High	No
C8	3.36	−4.48	−5.67	0.55	3.38	High	No
C9	3.59	−4.85	−5.83	0.55	3.53	High	No
C10	3.27	−5.21	−5.68	0.55	3.42	High	No
C11	3.14	−5.59	−5.16	0.55	3.33	High	No
C12	2.88	−7.15	−6.08	0.55	3.27	High	No
C13	4.12	−6.78	−6.59	0.55	3.24	High	No
C14	3.29	−6.41	−6.58	0.55	3.64	Low	No
C15	2.28	−6.04	−6.25	0.55	3.56	Low	No
C16	2.27	−6.14	−6.79	0.55	3.3	High	No
C17	3.12	−7.53	−6.94	0.55	3.25	Low	No
C18	2.21	−7.13	−6.77	0.55	3.44	Low	No
C19	2.37	−5.75	−6.61	0.55	3.1	High	No
C20	2.58	−6.02	−6.44	0.55	3.12	High	No
C21	2.85	−6.76	−6.62	0.55	3.14	High	No
C22	3.19	−4.35	−7.14	0.55	3.24	High	No
C23	3.69	−4.25	−7.29	0.55	3.35	High	No
C24	2.72	−4.62	−7.12	0.55	3.71	High	No
C25	2.17	−4.99	−6.95	0.55	3.52	High	No
C26	2.87	−5.33	−6.79	0.55	3.43	High	No
C27	2.87	−3.95	−6.79	0.55	3.41	High	No

### 3.2 Docking Studies

**FIGURE 1:**
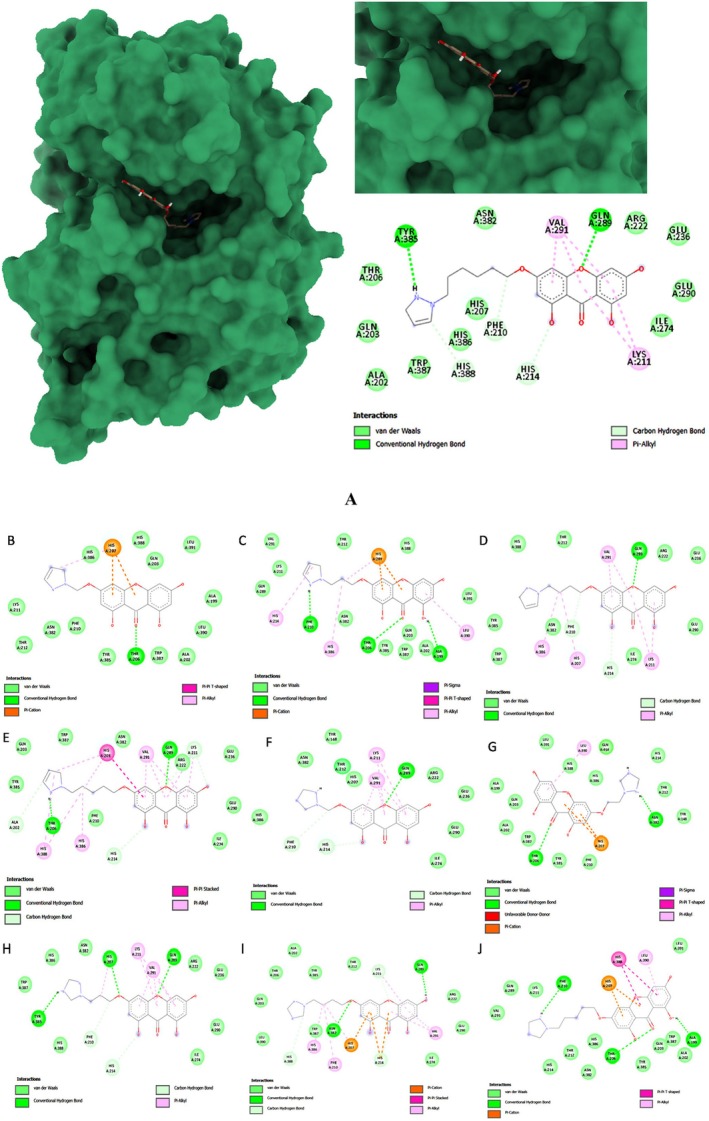
3D docked images of (A) A1 (B) A3 (C) A7 (D) A9 (E) A11 (F) A119 (G) A121 (H) A123 (I) A125 and (J) A127 against protein 1CX2.

The docking of the standard drug Indomethacin and the hybrid xanthone derivative was performed in Discovery Studio 2019 against COX‐2 for PDB‐ID: 1CX2. The binding energies of the ligands ranged from −8.7 to 8.0 kcal/mol. The highest binding affinity was observed with ligand A1having energy −8.7 kcal/mol (Figure 1). The amino acids that showed interaction with the ligands were Ala199, Thr206, Phe210, Gln289, and Tyr385. Table 4 given below indicates the list of synthesised ligands with their binding energies (Figure 1).

**TABLE 4:** Docking results for the 10 synthesised molecules with the highest binding affinities.

Compound code	Hydrogen bond interacting residues	Binding affinity (kcal/mol)
**A1(3‐(6′‐Pyrazolehexyloxy)‐1,6,8‐trihydroxy xanthone)**	Gln289, Tyr385	−8.7
**A3(3‐(1′‐Pyrazolemethyloxy)‐1,6,8‐trihydroxy xanthone)**	Tyr206	−8.2
**A7(3‐(3′‐Pyrazolepropyloxy)‐1,6,8‐trihydroxy xanthone)**	Ala199, Thr206, Phe210	−8.3
**A9(3‐(4′‐Pyrazolepropyloxy)‐1,6,8‐trihydroxy xanthone)**	Gln289	−8.1
**A11(3‐(5′‐Pyrazole pentyloxy)‐1,6,8‐trihydroxy xanthone)**	Thr206, Gln289	−8.3
**A119(3‐(1′‐(1,2,4‐Triazole)‐methyloxy)‐1,6,8‐trihydroxy xanthone)**	Gln289	−8.0
**A121(3‐(2′‐(1,2,4‐Triazole)‐ethyloxy)‐1,6,8‐trihydroxy xanthone)**	Thr206, Asn382	−8.4
**A123(3‐(3′‐(1,2,4‐Triazole)‐propyloxy)‐1,6,8‐trihydroxy xanthone)**	His207, Gln289, Tyr385	−8.4
**A125(3‐(4′‐(1,2,4‐Triazole)‐butyloxy)‐1,6,8‐trihydroxy xanthone)**	Gln289, Asn382	−8.1
**A127(3‐(5′(1,2,4‐Triazole)‐pentyloxy)‐1,6,8‐trihydroxy xanthone)**	Ala199, Thr206, Phe210	−8.0

#### 3.2.1 Molecular Dynamics Simulation

**FIGURE 2:**
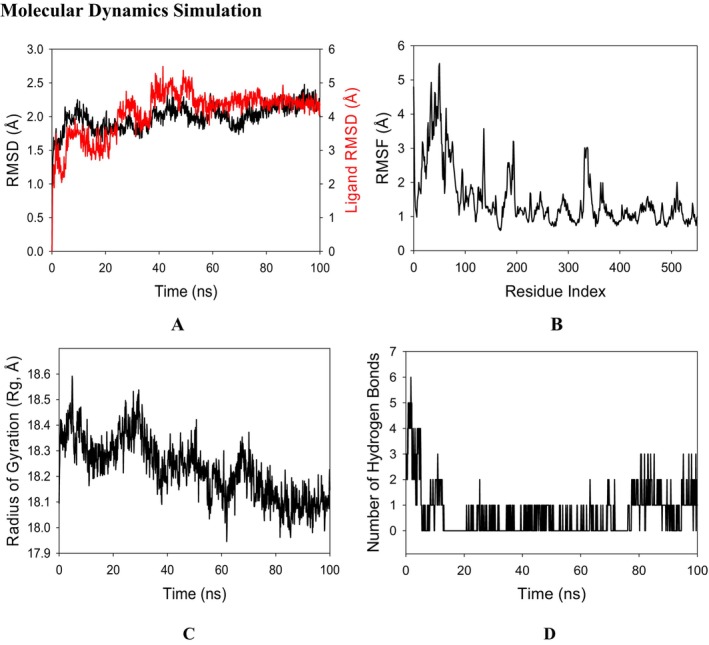
RMSD plots of Protein+Ligand where black plot is of protein and red plot is for ligand. (B) RMSF of Cα backbone of Protein bound to Ligand–ligand, (C) Radius of gyration (Rg) of Cα backbone of Protein bound to Ligand–ligand (D) Number of hydrogen bonds formed between portein and ligand.

Molecular dynamics and simulation (MD) studies were carried out in order to determine the stability and convergence of COX‐2 with ligand A1. Simulation of 100 ns displayed a stable conformation while comparing the root mean square deviation (RMSD) values. The RMSD of Cα‐backbone of COX‐2 bound to A1 exhibited a deviation of 2.1 Å, while the RMSD of the ligand exhibited 4.1 Å (Figure 2A). All the RMSD values are a little higher due to the protein conformation having a large number of loops and alpha helices. The stable RMSD plot during the simulation signifies good convergence and stable conformations. Therefore, it can be suggested that ligand A1 bound to COX‐2 is quite stable in complex due to the higher affinity of ligand A1. The plot for root mean square fluctuations (RMSF) displayed small spikes of fluctuation in COX‐2 bound to A1 where no significant spikes are observed except at 20–80 and 120 residue positions which may be due to the higher flexibility of the residues above 3 Å (Figure 2B). Most of the residues were less fluctuating during the entire 100 ns simulation (Figure 2B) indicating the stable amino acid conformations during the simulation time. Therefore, from the RMSF plots, it can be suggested that the protein structure is rigid during simulation in ligand‐bound conformations. The radius of gyration (Rg) is the measure of the compactness of the protein. Here in this study, COX‐2 bound to A1 displayed a lowering of the radius of gyration (Rg) from 18.4 to 18.1 Å (Figure 2C). Lowering of the gyration (Rg) indicates a highly compact orientation of the protein in the ligand‐bound state. The number of hydrogen bonds between the protein and ligand suggests significant interaction and stability of the complex. The number of hydrogen bonds between COX‐2 bound to A1 showed that a single number of hydrogen bonds, on average, was observed throughout the simulation time of 100 ns (Figure 2D), while at the beginning it reached up to six and at the last stages reached up to 3 hydrogen bonds. Therefore, MD simulation studies predicted a stable conformation of the protein‐ligand complex during 100 ns of simulation.

### 3.3 Chemistry

Out of all the designed 128 compounds, the 10 compounds that showed the best(lowest) affinity for binding energy in the docking study were taken for further synthesis. This included compounds having compound codes **A1(3‐(6′‐Pyrazolehexyloxy)‐1,6,8‐trihydroxy xanthone)**, **A3(3‐(1′‐Pyrazolemethyloxy)‐1,6,8‐trihydroxy xanthone)**, **A7(3‐(3′‐Pyrazolepropyloxy)‐1,6,8‐trihydroxy xanthone)**, **A9(3‐(4′‐Pyrazolepropyloxy)‐1,6,8‐trihydroxy xanthone)**, **A11(3‐(1′‐(1,2,4‐Triazole)‐methyloxy)‐1,6,8‐trihydroxy xanthone)**, **A119(3‐(1′‐(1,2,4‐Triazole)‐methyloxy)‐1,6,8‐trihydroxy xanthone)**, **A121(3‐(2′‐(1,2,4‐Triazole)‐ethyloxy)‐1,6,8‐trihydroxy xanthone)**, **A123(3‐(3′‐(1,2,4‐Triazole)‐propyloxy)‐1,6,8‐trihydroxy xanthone)**, **A125(3‐(4′‐(1,2,4‐Triazole)‐butyloxy)‐1,6,8‐trihydroxy xanthone) and A127(3‐(5′(1,2,4‐Triazole)‐pentyloxy)‐1,6,8‐trihydroxy xanthone)**. The synthetic scheme involves a three‐step reaction process, wherein the first step hydroxyl xanthone is produced (Scheme 1). The first step is the reaction of 2,4,6‐trihydroxy benzoic acid (1) with phloroglucinol (2) to give 1,3,6,8‐tetrahydroxy xanthone (3). Following this, there is alkoxy (4) substitution of the hydroxyxanthone derivative, and at last in the alkoxy substituted hydroxyxanthone (5) derivative is reacted with the heterocyclic compound (6) in order to give the end product (7). In Table 5 given below is the list of compounds synthesised along with the substitution on them. The structures of the synthesised compounds are confirmed by FTIR spectra, 1H and 13C NMR spectra, and mass spectra.

**TABLE 5:** List of the Ligands that were Synthesised and their Substitutes.

Sl no.	Synthesised ligand	Structure	Substituent
1	**A1 (3‐(6′‐Pyrazolehexyloxy)‐1,6,8‐trihydroxy xanthone)**	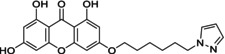	n‐C_6_H_12_; R‐C_3_H_4_N_2_
2	**A3 (3‐(1′‐Pyrazolemethyloxy)‐1,6,8‐trihydroxy xanthone)**	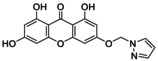	n‐CH_2_; R‐C_3_H_4_N_2_
3	**A7 (3‐(3′‐Pyrazolepropyloxy)‐1,6,8‐trihydroxy xanthone)**	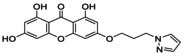	n‐C_3_H_6_; R‐C_3_H_4_N_2_
4	**A9 (3‐(4′‐Pyrazolepropyloxy)‐1,6,8‐trihydroxy xanthone)**	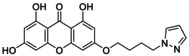	n‐C_4_H_8_; R‐C_3_H_4_N_2_
5	**A11(3‐(5′‐Pyrazole pentyloxy)‐1,6,8‐trihydroxy xanthone)**	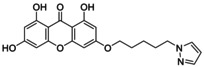	n‐C_5_H_10_; R‐C_3_H_4_N_2_
6	**A119 (3‐(1′‐(1,2,4‐Triazole)‐methyloxy)‐1,6,8‐trihydroxy xanthone)**	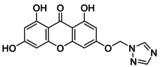	n‐CH_2_; R‐C_2_H_3_N_3_
7	**A121 (3‐(2′‐(1,2,4‐Triazole)‐ethyloxy)‐1,6,8‐trihydroxy xanthone)**	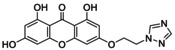	n‐C_2_H_4_; R‐C_2_H_3_N_3_
8	**A123 (3‐(3′‐(1,2,4‐Triazole)‐propyloxy)‐1,6,8‐trihydroxy xanthone)**	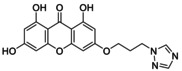	n‐C_3_H_6_; R‐C_2_H_3_N_3_
9	**A125 (3‐(4′‐(1,2,4‐Triazole)‐butyloxy)‐1,6,8‐trihydroxy xanthone)**	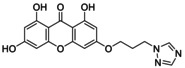	n‐C_4_H_8_; R‐C_2_H_3_N_3_
10	‐**A127 (3‐(5′(1,2,4‐Triazole)‐pentyloxy)‐1,6,8‐trihydroxy xanthone)**	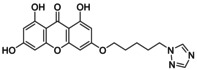	n‐C_5_H_10_; R‐C_2_H_3_N_3_

#### 3.3.1 Physicochemical Characterisation

##### 3.3.1.1 3‐(6′‐Pyrazolehexyloxy)‐1,6,8‐Trihydroxy Xanthone (C_22_H_22_N_2_O_6_ ) (Compound A1)

Dark brown solid; % yield 72.5; M.P. 176°C–181°C; *R*_f_ − 0.7; UV λ_max_ (Water): 268.80 nm; FTIR (cm^−1^): 3872.20, 3200.32 (O‐H str., phenolic); 2935.04, 2207.59 (C‐H str., alkane); 1616.67 (C=N str.sec. amine); 1392.29, 1359.28 (O‐H bend., alcohol); 1185.95 (C‐N str., sec. amine); 1091.70, 1048.81 (C‐O str., primary alcohol); 991.83, 824.15 (C=C bend. aromatic); ^1^HNMR (500mHz) DMSO, δ(ppm); 5.35 (aromatic C‐OH), 7.29 (‐CH of 1‐pyrazole), 6.39 (‐CH of 1‐benzene), 4.06 (‐CH_2_ of methylene); ^13^CNMR (500mHz) DMSO, δ(ppm); 139.6 (‐CH of pyrazole), 154.5 (‐C of 1‐benzene), 182.1 (‐C of carbonyl), 68.7 (‐CH_2_ of aliphatic), 27.1 (‐CH_2_ aliphatic), 96.4 (‐CH of 1‐benzene);MASS(m/z %): 395.05.

##### 3.3.1.2 3‐(1′‐Pyrazolemethyloxy)‐1,6,8‐Trihydroxy Xanthone (C_17_H_12_N_2_O_6_ ) (Compound A3)

Dark brown semi‐solid; % yield 71; M.P. 177°C–182°C;*R*_f_ − 0.75; UV λmax (Water): 265.80 nm; FTIR (cm^−1^): 3869.85, 3805.66, 3353.39 (O‐H str., phenolic); 2166.70 (C‐H str., alkane); 1623.65 (C=N str.sec. amine); 1371.42 (O‐H bend., alcohol); 1179.07 (C‐N str., sec. amine); 1088.16, 1045.82 (C‐O str., primary alcohol); 989.54 (C=C bend. aromatic); ^1H^NMR (500mHz) DMSO, δ(ppm); 5.35 (aromatic C‐OH), 6.22 (‐CH of 1‐pyrazole), 6.39 (‐CH of 1‐benzene), 6.36 (‐CH of 1‐benzene), 6.38 (‐CH_2_ of methylene);^13C^NMR (500mHz) DMSO, δ(ppm); 139.6 (‐CH of pyrazole), 129.6 (‐CH of pyrazole), 154.9 (‐C of 1‐benzene), 158.4 (‐C of 1‐benzene), 182.1 (‐C of carbonyl), 93.2 (‐CH of 1‐benzene), 90.0 (‐CH of 1‐benzene);MASS (m/z %): 361.12.

##### 3.3.1.3 3‐(3′‐Pyrazolepropyloxy)‐1,6,8‐Trihydroxy Xanthone (C_19_H_16_N_2_O_6_ ) (Compound A7)

Dark brown semi‐solid; % yield 81.5; M.P. 172°C–178°C; *R*_f_ − 0.6; UV λ_max_ (Water): 269.40 nm; FTIR (cm^−1^): 3355.95 (O‐H str., phenolic); 2164.92 (C‐H str., alkane); 1745.55 (C=O str., ketone); 1638.51(C=N str.sec. amine); 1525.31(C=C str., cyclic alkene); 1488.17 (C‐H bend. alkane); 1350.90 (O‐H bend., alcohol); 1279.49 (C‐O str., aromatic ester); 1181.27 (C‐N str., sec. amine); 1046.58 (C‐O str., primary alcohol); 974.21, 832.15 (C=C bend. aromatic);^1H^NMR (500mHz) DMSO, δ(ppm); 5.35 (aromatic C‐OH), 6.22 (‐CH of 1‐pyrazole), 6.39 (‐CH of 1‐benzene), 6.36 (‐CH of 1‐benzene), 6.38 (‐CH_2_ of methylene);^13C^NMR (500mHz) DMSO, δ(ppm); 139.6 (‐CH of pyrazole), 129.6 (‐CH of pyrazole), 154.9 (‐C of 1‐benzene), 158.4 (‐C of 1‐benzene), 182.1 (‐C of carbonyl), 93.2 (‐CH of 1‐benzene), 90.0 (‐CH of 1‐benzene);MASS (m/z %): 389.45.

##### 3.3.1.4 3‐(4′‐Pyrazolepropyloxy)‐1,6,8‐Trihydroxy Xanthone (C_20_H_18_N_2_O_6_ ) (Compound A9)

Dark brown solid; % yield 70.5; M.P. 165°C–170°C; *R*_f_ − 0.7; UV λ_max_ (Water): 268.60 nm; FTIR (cm^−1^): 3802.22, 3305.17 (O‐H str., phenolic); 2610.26, 2210.24 (C‐H str., alkane); 1595.98 (C=C str., cyclic alkene); 1391.66, 1354.96 (O‐H bend., alcohol); 1187.83 (C‐N str., sec. amine); 1046.89 (C‐O str., primary alcohol); 927.23, 873.56, 826.85 (C=C bend. aromatic); NMR; ^1H^NMR (500mHz) DMSO, δ(ppm); 5.35 (aromatic C‐OH), 7.29 (‐CH of 1‐pyrazole), 6.39 (‐CH of 1‐benzene), 4.06 (‐CH_2_ of methylene) ^13C^NMR (500mHz) DMSO, δ(ppm); 139.6 (‐CH of pyrazole), 154.5 (‐C of 1‐benzene), 182.1 (‐C of carbonyl), 68.7 (‐CH_2_ of aliphatic), 27.1 (‐CH_2_ aliphatic), 96.4 (‐CH of 1‐benzene);MASS (m/z %): 396.23.

##### 3.3.1.5 3‐(5′‐Pyrazole Pentyloxy)‐1,6,8‐Trihydroxy Xanthone (C_21_H_20_N_2_O_6_ ) (Compound A11)

Brown solid; % yield 85; M.P. 155°C–160°C; *R*_f_ − 0.78; UV λ_max_ (Water): 259.40 nm; FTIR (cm^−1^): 3814.30, 3327.47 (O‐H str., phenolic); 1599.31 (C=C str., cyclic alkene), 1356.84 (O‐H bend., alcohol); 1184.14 (C‐N str., sec. amine); 1087.09, 1045.35 (C‐O str., primary alcohol); 987.65, 826.65 (C=C bend. aromatic);^1H^NMR (500mHz) DMSO, δ(ppm); 5.35 (aromatic C‐OH), 6.22 (‐CH of 1‐pyrazole), 6.39 (‐CH of 1‐benzene), 6.36 (‐CH of 1‐benzene), 6.38 (‐CH_2_ of methylene);^13C^NMR (500mHz) DMSO, δ(ppm); 139.6 (‐CH of pyrazole), 129.6 (‐CH of pyrazole), 154.9 (‐C of 1‐benzene), 158.4 (‐C of 1‐benzene), 182.1 (‐C of carbonyl), 93.2 (‐CH of 1‐benzene), 90.0 (‐CH of 1‐benzene); MASS (m/z %): 408.09.

##### 3.3.1.6 3‐(1′‐(1,2,4‐Triazole)‐Methyloxy)‐1,6,8‐Trihydroxy Xanthone (C_16_H_11_N_3_O_6_ ) (Compound A119)

Dark brown solid; % yield 77.5; M.P. 169°C–173°C; *R*_f_ − 0.7; UV λ_max_ (Water): 266.80 nm; FTIR (cm^−1^): 3798.81, 3419.00, 3123.10 (O‐H str., phenolic); 2356.54 (C‐H str., alkane); 1914.34 (C‐H bend. Aromatic compound); 1749.86 (C=O str., ketone); 1642.50 (C=N str.sec. amine); 1518.86 (C=C str., cyclic alkene); 1420.57 (C‐H bend. alkane); 1344.33 (O‐H bend., alcohol); 1276.54 (C‐O str., aromatic ester); 1159.13 (C‐N str., sec. amine); 1044.42 (C‐O str., primary alcohol); 956.88, 874.25 (C=C bend. aromatic); ^1H^NMR (500mHz) DMSO, δ(ppm); 5.35 (aromatic C‐OH), 7.29 (‐CH of 1‐pyrazole), 6.39 (‐CH of 1‐benzene), 4.06 (‐CH_2_ of methylene); ^13C^NMR (500mHz) DMSO, δ(ppm); 139.6 (‐CH of pyrazole), 129.6 (‐CH of pyrazole), 154.9 (‐C of 1‐benzene), 158.4 (‐C of 1‐benzene), 182.1 (‐C of carbonyl), 93.2 (‐CH of 1‐benzene), 90.0 (‐CH of 1‐benzene);MASS (m/z %): 372.69.

##### 3.3.1.7 3‐(2′‐(1,2,4‐Triazole)‐Ethyloxy)‐1,6,8‐Trihydroxy Xanthone (C_17_H_13_N_3_O_6_ ) (Compound A121)

Brownish yellow semi‐solid; % yield 79; M.P. 164°C–170°C; *R*_f_ − 0.76; UV λ_max_ (Water): 263.20 nm; FTIR (cm^−1^): 3804.82, 3374.37 (O‐H str., phenolic); 2125.76 (C‐H str., alkane); 1640.68 (C=N str.sec. amine); 1486.88 (C‐H bend. alkane); 1370.14 (O‐H bend., alcohol); 1279.37 (C‐O str., aromatic ester); 1182.31 (C‐N str., sec. amine); 1047.09 (C‐O str., primary alcohol); 973.84 (C=C bend. aromatic); ^1H^NMR (500mHz) DMSO, δ(ppm); 5.35 (aromatic C‐OH), 7.29 (‐CH of 1‐pyrazole), 6.39 (‐CH of 1‐benzene), 4.06 (‐CH_2_ of methylene); ^13C^NMR (500mHz) DMSO, δ(ppm); 139.6 (‐CH of pyrazole), 154.5 (‐C of 1‐benzene), 182.1 (‐C of carbonyl), 68.7 (‐CH_2_ of aliphatic), 27.1 (‐CH_2_ aliphatic), 96.4 (‐CH of 1‐benzene); MASS (m/z %): 369.35.

##### 3.3.1.8 3‐(3′‐(1,2,4‐Triazole)‐Propyloxy)‐1,6,8‐Trihydroxy Xanthone (C_18_H_15_N_3_O_6_ ) (Compound A123)

Brownish yellow semi‐solid; % yield 75; M.P. 179°C–185°C; *R*_f_ − 0.7; UV λ_max_ (Water): 265.00 nm; FTIR (cm^−1^): 3801.97, 3785.51, 3127.06 (O‐H str., phenolic); 2975.73 (C‐H str., alkane); 1841.75 (C‐H bend. Aromatic compound); 1747.17 (C=O str., ketone); 1602.65 (C=N str.sec. amine); 1517.41 (C=C str., cyclic alkene); 1419.25 (C‐H bend. alkane); 1343.48 (O‐H bend., alcohol); 1278.66 (C‐O str., aromatic ester); 1159.80 (C‐N str., sec. amine); 1045.32 (C‐O str., primary alcohol); 970.17, 849.50 (C=C bend. aromatic); ^1H^NMR (500mHz) DMSO, δ(ppm); 5.35 (aromatic C‐OH), 6.22 (‐CH of 1‐pyrazole), 6.39 (‐CH of 1‐benzene), 6.36 (‐CH of 1‐benzene), 6.38 (‐CH_2_ of methylene);^13C^NMR (500mHz) DMSO, δ(ppm); 139.6 (‐CH of pyrazole), 154.5 (‐C of 1‐benzene), 182.1 (‐C of carbonyl), 68.7 (‐CH_2_ of aliphatic), 27.1 (‐CH_2_ aliphatic), 96.4 (‐CH of 1‐benzene);MASS (m/z %): 383.13.

##### 3.3.1.9 3‐(4′‐(1,2,4‐Triazole)‐Butyloxy)‐1,6,8‐Trihydroxy Xanthone (C_19_H_16_N_3_O_6_ ) (Compound 125)

Brown semi‐solid; % yield 79; M.P. 164°C–169°C; *R*_f_ − 0.78; UV λ_max_ (Water): 265.20 nm; FTIR (cm^−1^): 3808.12, 3743.51, 3251.92 (O‐H str., phenolic); 2629.01, 2399.37 (C‐H str., alkane); 1910.30 (C‐H bend. Aromatic compound); 1746.48 (C=O str., ketone); 1644.74 (C=N str.sec. amine); 1521.76 (C=C str., cyclic alkene); 1349.75 (O‐H bend., alcohol); 1279.70 (C‐O str., aromatic ester); 1182.60 (C‐N str., sec. amine); 1046.78, 1003.55 (C‐O str., primary alcohol); 960.46, 870.36, 832.25 (C=C bend. aromatic); ^1H^NMR (500mHz) DMSO, δ(ppm); 5.35 (‐OH, aromatic C‐OH), 7.29 (‐CH of 1‐pyrazole), 6.39 (‐CH of 1‐benzene); ^13C^NMR (500mHz) DMSO, δ(ppm); 139.6 (‐CH of pyrazole), 129.6 (‐CH of pyrazole), 154.9 (‐C of 1‐benzene), 158.4 (‐C of 1‐benzene), 182.1 (‐C of carbonyl), 93.2 (‐CH of 1‐benzene), 90.0 (‐CH of 1‐benzene); MASS (m/z %): 397.48.

##### 3.3.1.10 3‐(5′(1,2,4‐Triazole)‐Pentyloxy)‐1,6,8‐Trihydroxy Xanthone (C_20_H_19_N_3_O_6_ ) (Compound 127)

Brown semi‐solid; % yield 84.5; M.P. 158°C–163°C; *R*_f_ − 0.7; UV λ_max_ (Water): 264.80 nm; FTIR (cm^−1^): 3741.56, 3398.70 (O‐H str., phenolic); 2662.68, 2395.15, 2160.78 (C‐H str., alkane); 1745.00 (C=O str., ketone); 1637.78 (C=N str.sec. amine); 1521.10 (C=C str., cyclic alkene); 1487.51(C‐H bend. alkane); 1361.56 (O‐H bend., alcohol); 1280.25 (C‐O str., aromatic ester); 1047.47 (C‐O str., primary alcohol); 970.55, 874.29 (C=C bend. aromatic); ^1H^NMR (500mHz) DMSO, δ(ppm); 5.35 (aromatic C‐OH), 7.29 (‐CH of 1‐pyrazole), 6.39 (‐CH of 1‐benzene), 4.06 (‐CH_2_ of methylene); ^13C^NMR (500mHz) DMSO, δ(ppm); 139.6 (‐CH of pyrazole), 129.6 (‐CH of pyrazole), 154.9 (‐C of 1‐benzene), 158.4 (‐C of 1‐benzene), 182.1 (‐C of carbonyl), 93.2 (‐CH of 1‐benzene), 90.0 (‐CH of 1‐benzene);MASS (m/z %): 410.44.

### 3.4 In Vitro Anti‐Inflammatory Study

**FIGURE 3:**
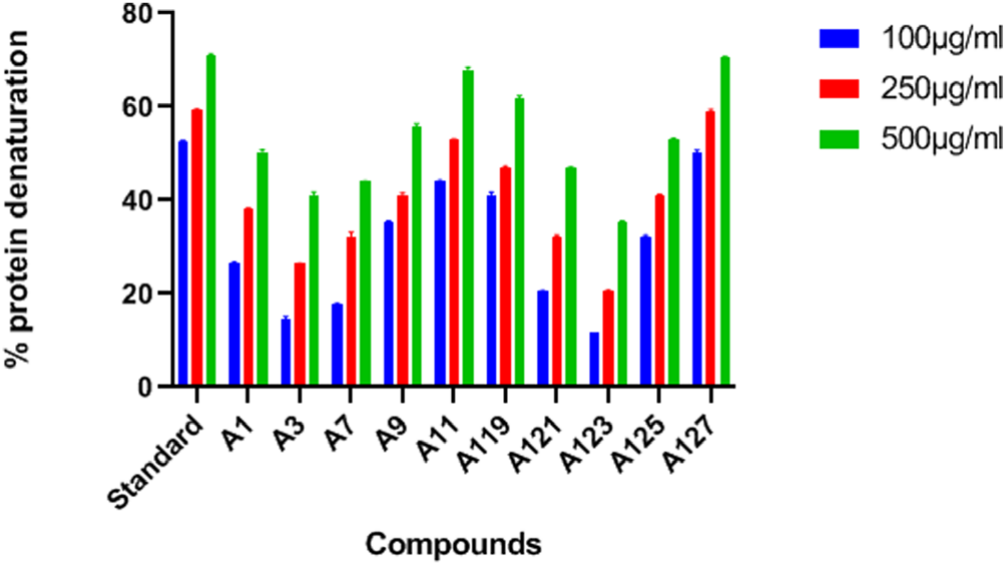
Graph representing % of protein denaturation by synthesised compounds.

The in vitro anti‐inflammatory activity of the synthesised ligands is studied by the Egg‐albumin Denaturation method. In this study, the protein present in the albumin loses the biological characteristic that is necessary for a protein molecule. This loss results in the denaturation of the protein which is accountable for the inflammation. Now for the study, the level to prevent the protein denaturation is carried out for the synthesised compounds at three different concentrations of 100, 250, and 500 μg/mL. The percentage of protein denaturation where maximum inhibition is shown by the compound A127**(3‐(5′(1,2,4‐Triazole)‐pentyloxy)‐1,6,8‐trihydroxy xanthone)** is 50%, 58.82%, and 70.58% respectively, which is followed by A11**(3‐(5′‐Pyrazole pentyloxy)‐1,6,8‐trihydroxy xanthone)** and A119**(3‐(1′‐(1,2,4‐Triazole)‐methyloxy)‐1,6,8‐trihydroxy xanthone)** as seen in the given Table 6 and Figure 3.

**TABLE 6:** Impact of in vitro protein denaturation on synthesised compounds: Egg Albumin Denaturation Method.

Group	Concentration (μg/mL)/% protein denaturation		
	100 μg/mL	250 μg/mL	500 μg/mL
Control			
**Standard**	52.55% ± 0.21	59.23% ± 0.32	71.13% ± 0.19
**A1(3‐(6′‐Pyrazolehexyloxy)‐1,6,8‐trihydroxy xanthone)**	26.47% ± 0.38	38.23% ± 0.1	50% ± 0.84
**A3(3‐(1′‐Pyrazolemethyloxy)‐1,6,8‐trihydroxy xanthone)**	14.7% ± 0.45	26.47% ± 0.1	41.17% ± 0.62
**A7(3‐(3′‐Pyrazolepropyloxy)‐1,6,8‐trihydroxy xanthone)**	17.64% ± 0.33	32.35% ± 0.84	44.11% ± 0.07
**A9(3‐(4′‐Pyrazolepropyloxy)‐1,6,8‐trihydroxy xanthone)**	35.29% ± 0.34	41.17% ± 0.35	55.8% ± 0.56
**A11(3‐(5′‐Pyrazole pentyloxy)‐1,6,8‐trihydroxy xanthone)**	44.11% ± 0.11	52.94% ± 0.1	67.64% ± 0.77
**A119(3‐(1′‐(1,2,4‐Triazole)‐methyloxy)‐1,6,8‐trihydroxy xanthone)**	41.17% ± 0.51	47.05% ± 0.22	61.82% ± 0.54
**A121(3‐(2′‐(1,2,4‐Triazole)‐ethyloxy)‐1,6,8‐trihydroxy xanthone**	20.58% ± 0.04	32.35% ± 0.21	47.05% ± 0.13
**A123(3‐(3′‐(1,2,4‐Triazole)‐propyloxy)‐1,6,8‐trihydroxy xanthone)**	11.76% ± 0.02	20.58% ± 0.1	35.29% ± 0.37
**A125(3‐(4′‐(1,2,4‐Triazole)‐butyloxy)‐1,6,8‐trihydroxy xanthone)**	32.35% ± 0.12	41.17% ± 0.09	52.94% ± 0.29
**A127(3‐(5′(1,2,4‐Triazole)‐pentyloxy)‐1,6,8‐trihydroxy xanthone)**	50% ± 0.76	58.82% ± 0.62	70.58% ± 0.19

### 3.5 In Vivo Anti‐Inflammatory Activity

**FIGURE 4:**
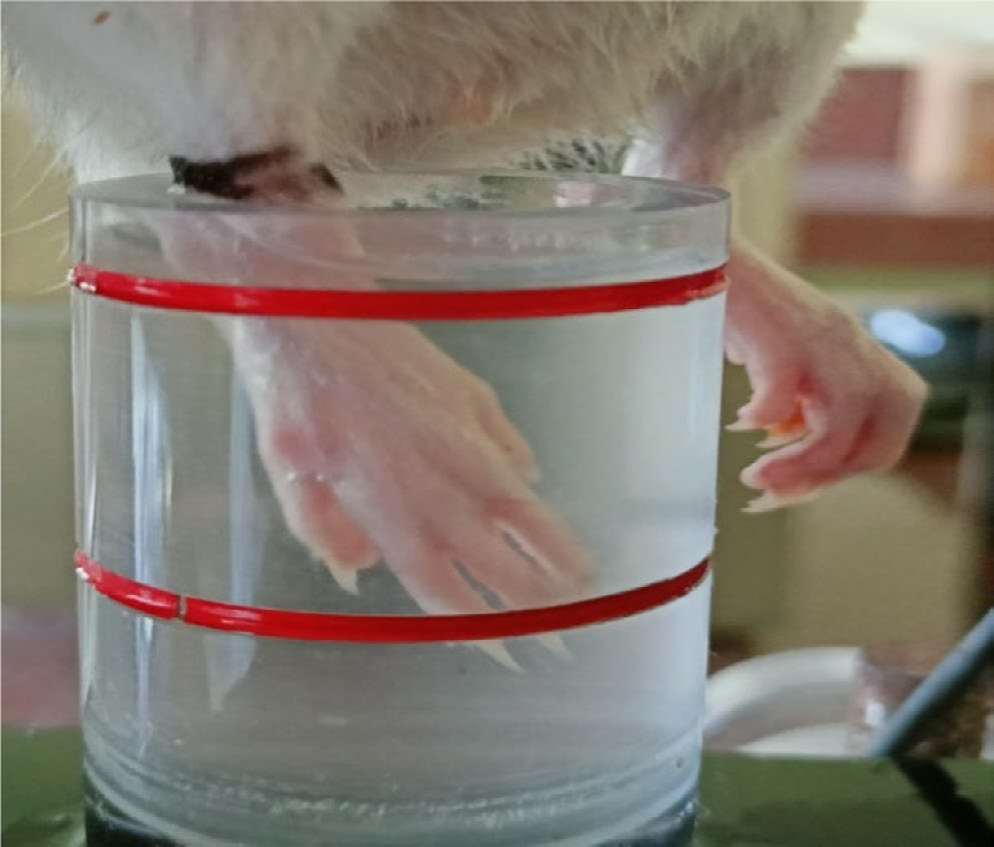
Carrageenan induced in the right hind paw.

**FIGURE 5:**
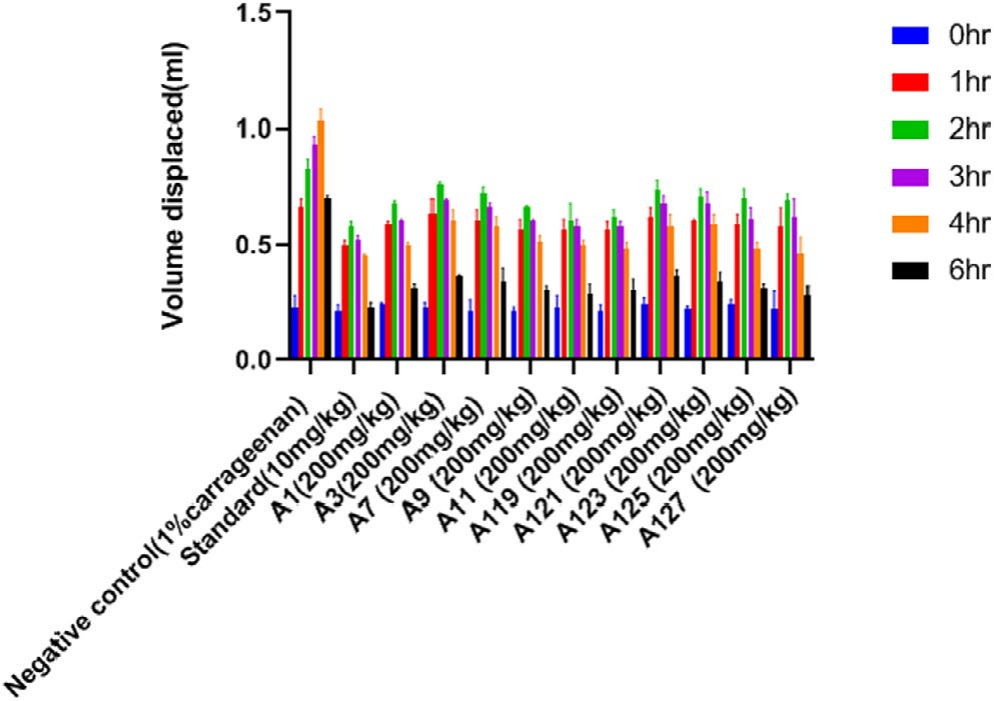
Graph representing the volume displacement by different synthesised compounds.

**FIGURE 6:**
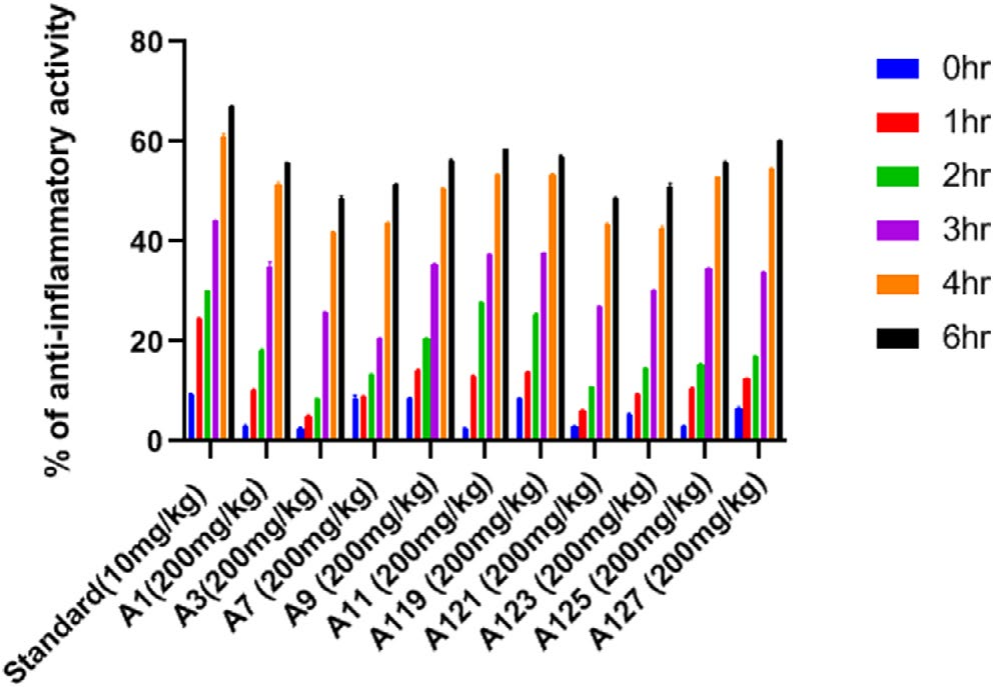
Graph representing the % of anti‐inflammatory effect of synthesised compounds.

The in vivo anti‐inflammatory activity is carried out by the Carrageenan‐induced paw edema method on the Wistar rats (Figure 4). All the 10 synthesised compounds as per acute toxicity studies at the highest dose were reported to not have any kind of toxicity. For these 10 compounds, the anti‐inflammatory activity was studied using the Carrageenan‐induced paw edema method. In this, the volume displaced is measured after inducing edema on the right hind paw. Table 7 consists of the result of the volume displaced after inducing Carrageenan and the oral administration of the synthesised compounds at a time interval of 0, 1, 2, 3, 4, and 6 h (Figure 5). Table 8 shows the result of percentage inhibition at different time intervals. The compound A127**(3‐(5′(1,2,4‐Triazole)‐pentyloxy)‐1,6,8‐trihydroxy xanthone)** showed the best activity in comparison with the standard drug. This was followed by A11**(3‐(5′‐Pyrazole pentyloxy)‐1,6,8‐trihydroxy xanthone)**, A119**(3‐(1′‐(1,2,4‐Triazole)‐methyloxy)‐1,6,8‐trihydroxy xanthone)**, A9 and A125**(3‐(4′‐(1,2,4‐Triazole)‐butyloxy)‐1,6,8‐trihydroxy xanthone)**. The hybrid compounds A127**(3‐(5′(1,2,4‐Triazole)‐pentyloxy)‐1,6,8‐trihydroxy xanthone)**, A11**(3‐(5′‐Pyrazole pentyloxy)‐1,6,8‐trihydroxy xanthone)**, A119**(3‐(1′‐(1,2,4‐Triazole)‐methyloxy)‐1,6,8‐trihydroxy xanthone)**, A9**(3‐(4′‐Pyrazolepropyloxy)‐1,6,8‐trihydroxy xanthone)**, and A125**(3‐(4′‐(1,2,4‐Triazole)‐butyloxy)‐1,6,8‐trihydroxy xanthone)** showed significant percentage inhibition of 60%, 58.57%, 57.14%, 56.24%, and 55.71% respectively at the dose of 200 mg/kg. The rest of the compounds were found less effective than the others. The serum level for the pro and anti‐inflammatory cytokine was measured for IL‐6, TNF‐α, IL‐4, and IL‐10 by the use of a commercial sandwich ELISA kit. Table 9 consists of the effect of the five synthesised compounds on the cytokine which gave the best result in comparison with the standard (Figure 6).

**TABLE 7:** Evaluation of synthesised hybrid‐xanthone derivatives by Carrageenan–Induced Paw Edema method in rats.

Group	Dose (mg/kg)	Volume displaced in mL					
		0 h	1 h	2 h	3 h	4 h	6 h
**Negative control**	1% Carrageenan soln	0.23 ± 0.05	0.66 ± 0.04	0.83 ± 0.04	0.93 ± 0.047	1.03 ± 0.05	0.70 ± 0.015
**Standard**	10 mg/kg	0.21 ± 0.03	0.5 ± 0.02	0.58 ± 0.026	0.52 ± 0.026	0.4 ± 0.015	0.23 ± 0.025
**A1(3‐(6′‐Pyrazolehexyloxy)‐1,6,8‐trihydroxy xanthone)**	200 mg/kg	0.24 ± 0.011**	0.59 ± 0.015	0.68 ± 0.015	0.60 ± 0.015	0.50 ± 0.017	0.31 ± 0.02
**A3(3‐(1′‐Pyrazolemethyloxy)‐1,6,8‐trihydroxy xanthone)**	200 mg/kg	0.23 ± 0.02**	0.63 ± 0.072	0.76 ± 0.017	0.69 ± 0.01	0.60 ± 0.005	0.36 ± 0.01
**A7(3‐(3′‐Pyrazolepropyloxy)‐1,6,8‐trihydroxy xanthone)**	200 mg/kg	0.21 ± 0.05**	0.60 ± 0.05	0.72 ± 0.03	0.66 ± 0.02	0.58 ± 0.04	0.34 ± 0.06
**A9(3‐(4’‐Pyrazolepropyloxy)‐1,6,8‐trihydroxy xanthone)**	200 mg/kg	0.21 ± 0.02**	0.57 ± 0.04	0.66 ± 0.011	0.60 ± 0.011	0.51 ± 0.03	0.30 ± 0.02
**A11(3‐(5’‐Pyrazole pentyloxy)‐1,6,8‐trihydroxy xanthone)**	200 mg/kg	0.23 ± 0.05**	0.57 ± 0.04	0.60 ± 0.08**	0.58 ± 0.03	0.5 ± 0.02	0.29 ± 0.04
**A119(3‐(1′‐(1,2,4‐Triazole)‐methyloxy)‐1,6,8‐trihydroxy xanthone)**	200 mg/kg	0.21 ± 0.03**	0.57 ± 0.03	0.62** ± 0.03	0.58 ± 0.02	0.48 ± 0.03	0.3 ± 0.05
**A121(3‐(2′‐(1,2,4‐Triazole)‐ethyloxy)‐1,6,8‐trihydroxy xanthone**	200 mg/kg	0.24 ± 0.03**	0.62 ± 0.04	0.74 ± 0.04	0.68 ± 0.03	0.58 ± 0.051	0.36 ± 0.035
**A123(3‐(3′‐(1,2,4‐Triazole)‐propyloxy)‐1,6,8‐trihydroxy xanthone)**	200 mg/kg	0.22 ± 0.015**	0.60 ± 0.011	0.71 ± 0.03	0.68 ± 0.05	0.59 ± 0.04	0.34 ± 0.04
**A125(3‐(4′‐(1,2,4‐Triazole)‐butyloxy)‐1,6,8‐trihydroxy xanthone)**	200 mg/kg	0.24 ± 0.02**	0.59 ± 0.04	0.7 ± 0.04	0.61 ± 0.05	0.48 ± 0.037	0.31 ± 0.02
**A127(3‐(5′(1,2,4‐Triazole)‐pentyloxy)‐1,6,8‐trihydroxy xanthone)**	200 mg/kg	0.22 ± 0.08**	0.58 ± 0.085	0.69 ± 0.03	0.62 ± 0.08	0.46 ± 0.07	0.28 ± 0.045**

*Note:* All the values are given as Mean ± SEM (*n* = 6), Standard vs. all group (**p < 0.01, *p < 0.05). Indomethacin treated group, Synthetic drug treated groups were compared with the Carrageenan treated control (Inflammatory control).

** *p* < 0.01: statistically very significant.

**TABLE 8:** Assessment of percentage inhibition of synthesised compounds at various time intervals.

Group	Dose	Percentage of anti‐inflammatory activity at different intervals (%)					
		0 h	1 h	2 h	3 h	4 h	6 h
**Standard**	10 mg/kg	9.47 ± 0.17	24.58 ± 0.12	30.12 ± 0.02	44.08 ± 0.15	61.16 ± 0.43	67.14 ± 0.12
**A1(3‐(6′‐Pyrazolehexyloxy)‐1,6,8‐trihydroxy xanthone)**	200 mg/kg	2.96 ± 0.31	10.1 ± 0.41	18.07 ± 0.51	35.16 ± 0.82	51.45 ± 0.36	55.71 ± 0.14
**A3(3‐(1′‐Pyrazolemethyloxy)‐1,6,8‐trihydroxy xanthone)**	200 mg/kg	2.54 ± 0.31	4.97 ± 0.42	8.43 ± 0.21	25.8 ± 0.19	41.74 ± 0.31	48.57 ± 0.52
**A7(3‐(3′‐Pyrazolepropyloxy)‐1,6,8‐trihydroxy xanthone)**	200 mg/kg	8.69 ± 0.51	9.04 ± 0.13	13.25 ± 0.22	20.48 ± 0.27	43.68 ± 0.18	51.42 ± 0.21
**A9(3‐(4′‐Pyrazolepropyloxy)‐1,6,8‐trihydroxy xanthone)**	200 mg/kg	8.47 ± 0.38	14.02 ± 0.41	20.48 ± 0.22	35.48 ± 0.13	50.48 ± 0.21	56.24 ± 0.32
**A11(3‐(5’‐Pyrazole pentyloxy)‐1,6,8‐trihydroxy xanthone)**	200 mg/kg	2.54 ± 0.19	13.12 ± 0.21	27.71 ± 0.22	37.39 ± 0.11	53.29 ± 0.27	58.57 ± 0.023
**A119(3‐(1′‐(1,2,4‐Triazole)‐methyloxy)‐1,6,8‐trihydroxy xanthone)**	200 mg/kg	8.47 ± 0.31	13.63 ± 0.41	25.3 ± 0.34	37.63 ± 0.16	53.39 ± 0.24	57.14 ± 0.21
**A121(3‐(2′‐(1,2,4‐Triazole)‐ethyloxy)‐1,6,8‐trihydroxy xanthone**	200 mg/kg	2.96 ± 0.21	6.06 ± 0.22	10.84 ± 0.13	26.88 ± 0.21	43.39 ± 0.31	48.57 ± 0.31
**A123(3‐(3′‐(1,2,4‐Triazole)‐propyloxy)‐1,6,8‐trihydroxy xanthone)**	200 mg/kg	5.5 ± 0.11	9.5 ± 0.12	14.45 ± 0.33	30.12 ± 0.21	42.71 ± 0.32	51 ± 0.72
**A125(3‐(4′‐(1,2,4‐Triazole)‐butyloxy)‐1,6,8‐trihydroxy xanthone)**	200 mg/kg	2.96 ± 0.28	10.55 ± 0.31	15.3 ± 0.31	34.4 ± 0.32	52.81 ± 0.11	55.71 ± 0.41
**A127(3‐(5′(1,2,4‐Triazole)‐pentyloxy)‐1,6,8‐trihydroxy xanthone)**	200 mg/kg	6.77 ± 0.21	12.51 ± 0.14	16.86 ± 0.32	33.76 ± 0.25	54.75 ± 0.04	60 ± 0.31

*Note:* All the values are given as Mean ± SEM (*n* = 6), Standard vs. all group. Indomethacin treated group, Synthetic drug treated groups were compared with the Carrageenan treated control (inflammatory control).

**TABLE 9:** Impact of synthesised compounds on cytokine expression.

Sl no.	Drug	Dose	Anti‐inflammatory cytokine factors		Pro‐inflammatory cytokine factors	
			Concentration of IL‐4 (pg/mL)	Concentration of IL‐10 (pg/mL)	Concentration of IL‐6 (pg/mL)	Concentration of TNF‐α (pg/mL)
1	**Negative control**	1% Carrageenan soln	15.61	12.19	70.12	125.00
2	**Standard**	10 mg/kg	50.07	39.12	19.03	10.05
3	**A1(3‐(6′‐Pyrazolehexyloxy)‐1,6,8‐trihydroxy xanthone)**	200 mg/kg	22.51	21.09	13.15	15.95
4	**A9(3‐(4′‐Pyrazolepropyloxy)‐1,6,8‐trihydroxy xanthone)**	200 mg/kg	32.11	17.9	20.11	12.02
5	**A11(3‐(5′‐Pyrazole pentyloxy)‐1,6,8‐trihydroxy xanthone)**	200 mg/kg	20.05	15.61	15.09	13.6
6	**A119(3‐(1′‐(1,2,4‐Triazole)‐methyloxy)‐1,6,8‐trihydroxy xanthone)**	200 mg/kg	37.91	19.51	12.01	17.78
7	**A127(3‐(5′(1,2,4‐Triazole)‐pentyloxy)‐1,6,8‐trihydroxy xanthone)**	200 mg/kg	48.51	37.51	12.51	10.12

### 3.6 SAR Studies of Xanthones

- Xanthones and hydroxyxanthones are bioactive compounds that exhibit significant anti‐inflammatory properties. Structurally, xanthones are tricyclic compounds (C13H8O2) composed of a dibenzo‐γ‐pyrone framework, while hydroxyxanthones refer to xanthones that contain hydroxyl groups (‐OH) at various positions on their core structure. These modifications are critical in influencing their biological activities, including anti‐inflammatory effects.
- The planar tricyclic scaffold of xanthones is essential for their anti‐inflammatory properties. The basic xanthone structure, with its conjugated double bonds and carbonyl group, allows interaction with various molecular targets involved in inflammation.
- However, the presence, number, and positions of substituents on the xanthone skeleton modulate their efficacy in inhibiting inflammatory pathways.
- Hydroxyl groups significantly impact the anti‐inflammatory potency of xanthones. Their position on the xanthone backbone, along with the presence of other functional groups like methoxy (‐OCH3), methyl (‐CH3), or glycosides, modulate their activity.
- Increased hydroxylation tends to enhance anti‐inflammatory activity. The hydroxyl groups contribute to the ability of xanthones to scavenge reactive oxygen species (ROS) and inhibit pro‐inflammatory mediators such as nitric oxide (NO) and cytokines. Hydroxylated xanthones also show increased interaction with molecular targets such as cyclooxygenase (COX) and nuclear factor‐kappa B (NF‐κB).
- The position of the hydroxyl group(s) on the xanthone scaffold plays a critical role in determining the extent of anti‐inflammatory activity. Hydroxylation at the 1st, 3rd, and 5th positions has been shown to enhance inhibitory effects on pro‐inflammatory enzymes like COX‐2 and 5‐lipoxygenase (5‐LOX).
- **1‐Hydroxyxanthone** and **3‐hydroxyxanthone** both are known for their strong inhibitory activity against COX‐2, a key enzyme involved in the inflammatory cascade.
- **5‐Hydroxyxanthone** exhibits potent antioxidant activity that supports its role in reducing inflammation through ROS scavenging.
- The presence of multiple hydroxyl groups, such as in **1,3‐dihydroxyxanthone** or **3,4,5‐trihydroxyxanthone**, tends to enhance their anti‐inflammatory and antioxidant activity. These hydroxylated derivatives are more effective in modulating key inflammatory pathways, including the inhibition of NF‐κB, inducible nitric oxide synthase (iNOS), and cytokine release.
- In addition to hydroxyl groups, methoxy (‐OCH3) and methyl (‐CH3) groups influence the anti‐inflammatory effects of xanthones.
- Methoxylated xanthones (e.g., **1,3,7‐trimethoxyxanthone**) tend to display moderate to strong anti‐inflammatory activity, depending on the substitution pattern. Methoxy groups can reduce the polarity of xanthones, enhancing their lipophilicity, which may facilitate better cell membrane penetration and interaction with intracellular targets.
- Methylated xanthones, such as **mangostin** (from *Garcinia mangostana* ), exhibit potent anti‐inflammatory activity by inhibiting the release of pro‐inflammatory cytokines (e.g., TNF‐α, IL‐6) and enzymes such as COX‐2. The introduction of methyl groups often enhances the bioavailability and tissue distribution of xanthones.
- Glycosylation of xanthones typically results in a reduction of their anti‐inflammatory activity. While glycosides of xanthones may possess other pharmacological properties like improved solubility, the aglycone (non‐sugar) form is generally more potent in anti‐inflammatory assays. Glycosylation can hinder the interaction of xanthones with molecular targets involved in inflammation, reducing their overall efficacy.
- The carbonyl group at position C9 of the xanthone backbone, along with hydroxyl groups, contributes to its nucleophilic and electrophilic interactions with various biomolecules. The electrophilic sites on the xanthone molecule enable it to form covalent bonds with thiol groups in target proteins, contributing to its ability to inhibit NF‐κB activation. NF‐κB is a critical transcription factor involved in the expression of numerous pro‐inflammatory genes.
- Hydroxylated xanthones downregulate the activation of NF‐κB, a transcription factor that regulates the expression of many inflammatory genes. By inhibiting NF‐κB, xanthones reduce the production of pro‐inflammatory cytokines, chemokines, and adhesion molecules.
- Some xanthones activate nuclear factor erythroid 2‐related factor 2 (Nrf2), a transcription factor that regulates antioxidant responses. Activation of the Nrf2 pathway leads to the upregulation of antioxidant enzymes, providing further protection against inflammation driven by oxidative stress.

### 3.7 QSAR Studies

**FIGURE 7:**
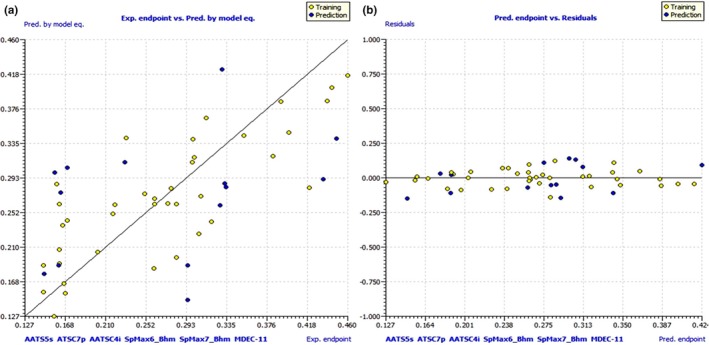
(a) Graph of experimental vs. predicted pIC50 values for the model; (b) Williams plot for themodel.

Despite the relatively small size of the dataset used in this investigation, a vast chemical landscape has been covered thanks to the presence of a wide variety of molecular scaffolds, functional groups, substituents, and rings (including but not limited to nonaromatic, aromatic, heteroaromatic, fused rings, spiro compounds, and so on). Hence, the QSAR models developed draw only from the subset of the dataset that was split. The QSAR models include a sufficient number of chemical descriptors, as shown by fitting parameters with values substantially above the permitted threshold values of R^2^, R^2^ adj, CCC_tr_, etc. The statistical reliability of the QSAR models is guaranteed by values of internal validation parameters like Q^2^ LOO, Q^2^ LMO, etc. (Figure 7a,b). High values of the external validation parameters R^2^ext, Q^2^‐Fn, etc. indicate that models are externally predictable. Model application domains are supported by Williams plot (Figure 7a,b). It is very unlikely that the QSAR models were developed by chance due to the achievement of authorised threshold values for numerous parameters and the poor correlation among the chemical descriptors. This evidence substantiates statistical robustness and good external predictability of these models.

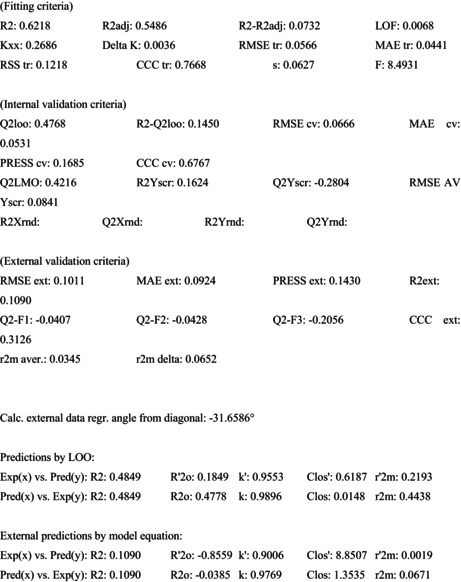

## 4 QSAR Model

_**P**_ **IC**_**50**_ = 1.8889 (± 0.4737) + 0.1037 (± 0.0246) × **AATS5S** + 0.0372 (± 0.0158) × **ATSC7p** + 0.4940 (± 0.1369) × **ATSC4i** + 0.4878 (± 0.1217) × **SpMax6_Bhm** + −1.2341 (± 0.2361) × **SpMax7_Bhm** + 0.4713(± 0.1149) × **MDEC‐11**.

**AATS5S—**autocorrelation of lag 5 weighted by intrinsic state.

**ATSC7p—**autocorrelation of lag 7 weighted by polarizability.

**ATSC4i—**autocorrelation of lag 4 weighted by ionisation potential.

**SpMax6_Bhm‐**occurrence of aromatic carbon atoms within six bonds from the sp2 hybridised oxygen atom.

**SpMax7_Bhm‐**occurrence of sp3 hybridised nitrogen atom within seven bonds from the donor atom.

**MDEC‐11‐** molecular distance edge between primary C and primary C.

Although for both these models, values of almost all the statistical parameters related to fitting criteria and internal validation are essentially the same, the differences in values of statistical parameters related to external validation, that is, *R* ^2^ ext. and *Q* ^2^‐Fn are noteworthy. Among the reported QSAR studies of COXs, the particularly recent work has been done using the dataset of 67 inhibitors (*R* ^2^ = 0.6218, *Q* ^2^ = 0.4768 and *R* ^2^ pred = 0.5846). The QSAR models developed in the present work are based on the dataset of a relatively large number of compounds with various different scaffolds and a large number of pharmacophoric features that have increased the scope of applicability of these models. Subjective feature selection provided some simple molecular descriptors; those are reflected in the QSAR models. Values of these molecular descriptors can be easily modified by introducing some simple constitutional and structural alterations to bring about optimisation.

### 4.1 QSAR Based Virtual Screening

Virtual screening of the most potent synthesised ligand molecules against the anti‐inflammatory target protein was determined and established in Table 10.

**TABLE 10:** Prediction of best molecule based on predicted IC50 values in QSAR based virtual screening.

Sl. No	Compound name	_P_ic50	IC50 in	Inference
1	3‐(2‐(1H‐1,2,4‐triazol‐1‐yl)ethoxy)‐1,6,8‐trihydroxy‐9H‐xanthen‐9‐one	6.38	416.869	
2	3‐(3‐(1H‐1,2,4‐triazol‐1‐yl)propoxy)‐1,6,8‐trihydroxy‐9H‐xanthen‐9‐one	6.031	931.108	
3	3‐(3‐(1H‐pyrazol‐1‐yl)propoxy)‐1,6,8‐trihydroxy‐9H‐xanthen‐9‐one	6.031	931.108	
4	3‐(4‐(1H‐pyrazol‐1‐yl)butoxy)‐1,6,8‐trihydroxy‐9H‐xanthen‐9‐one	7.401	39.719	MOST ACTIVE MOLECULE
5	3‐((1H‐1,2,4‐triazol‐1‐yl)methoxy)‐1,6,8‐trihydroxy‐9H‐xanthen‐9‐one	6.853	140.281	
6	3‐((1H‐pyrazol‐1‐yl)methoxy)‐1,6,8‐trihydroxy‐9H‐xanthen‐9‐one	5.114	7691.304	
7	3‐((5‐(1H‐1,2,4‐triazol‐1‐yl)pentyl)oxy)‐1,6,8‐trihydroxy‐9H‐xanthen‐9‐one	5.757	1749.847	
8	3‐((5‐(1H‐pyrazol‐1‐yl)pentyl)oxy)‐1,6,8‐trihydroxy‐9H‐xanthen‐9‐one	5.209	6180.164	
9	3‐((6‐(1H‐pyrazol‐1‐yl)hexyl)oxy)‐1,6,8‐trihydroxy‐9H‐xanthen‐9‐one	5.208	6350.120	

From the above Table 10 it has been concluded that the most active molecule with the lowest IC50 value is the most effective, and the value was found to be 39.719 nM for 3‐(4‐(1H‐pyrazol‐1‐yl)butoxy)‐1,6,8‐trihydroxy‐9H‐xanthen‐9‐one. However, the IC50 values used for calculating pIC50 values are experimental and sourced from the chEMBL database. This will help contextualise the pIC50 values in terms of their origin.

## 5 Discussion

The design and development of xanthone hybrids for potent anti‐inflammatory effects focus on enhancing the pharmacological properties of xanthone, a naturally occurring scaffold known for its diverse biological activities, including anti‐inflammatory, antioxidant, and anticancer effects. Xanthones are chemically versatile, allowing for structural modifications that can optimise their anti‐inflammatory potential. In this research, the synthesis of xanthone hybrids involves introducing various functional groups or conjugating the xanthone core with other bioactive moieties to improve its efficacy and selectivity towards inflammation‐related targets. The synthetic strategies aim to enhance solubility, bioavailability, and metabolic stability while maintaining or improving the core xanthone's anti‐inflammatory activity.

Structure–activity relationship (SAR) studies play a crucial role in this research by evaluating the impact of specific structural modifications on the biological activity of the synthesised xanthone hybrids. Through SAR analysis, researchers identify the essential pharmacophoric features and the influence of substituents on the anti‐inflammatory properties. This includes investigating the effects of electron‐donating or electron‐withdrawing groups, the nature and position of substitutions on the xanthone ring, and the overall molecular geometry. These findings allow for a rational design approach to further modify and optimise xanthone derivatives for better interaction with molecular targets such as COX enzymes, cytokines, or NF‐κB pathways involved in inflammation.

The anti‐inflammatory efficacy of the xanthone hybrids is evaluated through in vitro and in vivo models. In vitro assays assess the compounds' ability to inhibit the production of pro‐inflammatory mediators like nitric oxide (NO), prostaglandins, and inflammatory cytokines such as TNF‐α and IL‐6. However, in vivo studies, often conducted in animal models of inflammation, help validate the therapeutic potential of these compounds by evaluating their ability to reduce inflammation and related symptoms. The overall results demonstrate that well‐designed xanthone hybrids have promising anti‐inflammatory activity, making them potential candidates for the development of novel anti‐inflammatory drugs with improved efficacy and fewer side effects.

## 6 Conclusion

The study underscores the potential of xanthone‐heterocycle hybrids as a new class of anti‐inflammatory agents. These hybrid compounds, particularly A127, demonstrated superior efficacy in both in vitro and in vivo models by effectively inhibiting key inflammatory mediators, reducing protein denaturation, and alleviating paw edema. Their strong anti‐inflammatory effects, combined with a favourable safety and toxicity profile, make them promising candidates for further development as COX‐2 inhibitors and novel therapeutic agents for treating inflammatory disorders. Further, the results have promising industrial applications, particularly in the pharmaceutical sector for the development of novel anti‐inflammatory drugs. These xanthone‐heterocycle hybrids could be further developed into prescription medications for treating inflammation‐related conditions, such as arthritis, autoimmune disorders, and other chronic inflammatory diseases. Moreover, the favourable safety profile of the compounds makes them viable candidates for preclinical and clinical development. Pharmaceutical companies could explore these hybrids as alternatives to existing non‐steroidal anti‐inflammatory drugs (NSAIDs), particularly for patients requiring long‐term anti‐inflammatory therapy with fewer side effects.

Moving forward, specific research directions should focus on optimising the molecular structure of xanthone‐based hybrids to develop more selective and potent COX‐2 inhibitors. This could involve refining the core xanthone scaffold or modifying the attached heterocyclic moieties to increase selectivity for COX‐2 over COX‐1, thereby minimising gastrointestinal side effects typically associated with non‐selective NSAIDs. Structure–activity relationship (SAR) studies can further pinpoint the most effective functional groups responsible for high COX‐2 selectivity and anti‐inflammatory potency.

Moreover, exploration of other therapeutic applications for these compounds is a promising avenue. Xanthone derivatives have been shown to exhibit diverse pharmacological activities, including antioxidant, antimicrobial, and anticancer effects. Therefore, future studies could investigate the potential of xanthone –heterocycle hybrids in treating other inflammation‐related conditions, such as neurodegenerative diseases (e.g., Alzheimer's and Parkinson's), cardiovascular diseases, or cancers driven by chronic inflammation.

Moreover, further in‐depth pharmacokinetic and pharmacodynamic studies, as well as long‐term toxicity assessments, are necessary to evaluate the clinical viability of these compounds. Expanding the in vivo research to include chronic models of inflammation and testing in higher‐order animal species will help determine their therapeutic potential and safety profiles in a more complex biological context. Lastly, the development of drug delivery systems that enhance the bioavailability of xanthone‐based hybrids, such as nanoparticle‐based formulations or targeted delivery approaches, could significantly improve their therapeutic efficacy and application in clinical settings.

Thus, future research should focus not only on improving the selectivity and safety of these hybrid compounds but also on exploring their full therapeutic potential in various diseases where inflammation plays a central role. These efforts may lead to the discovery of novel, highly effective anti‐inflammatory drugs with wide‐reaching clinical applications.

## Author Contributions

**Shreyasi Karmakar:** conceptualization (lead), data curation (lead), writing – original draft (lead). **Riya Saikia:** conceptualization (equal), data curation (equal), formal analysis (equal), investigation (equal), methodology (equal), validation (equal), writing – original draft (equal). **Aparoop Das:** conceptualization (equal), project administration (equal), supervision (equal), validation (equal), writing – review and editing (equal). **Kalyani Pathak:** data curation (equal), formal analysis (equal), writing – review and editing (equal). **Padmashree Das:** formal analysis (equal), investigation (equal), methodology (equal), writing – original draft (equal). **Biman Bhuyan:** data curation (equal), software (equal), supervision (equal). **Taha Alqahtani:** data curation (equal), investigation (equal), writing – review and editing (equal). **Humood Al Shmrany:** data curation (equal), formal analysis (equal), writing – review and editing (equal). **Bikram Dhara:** validation (equal), visualization (equal), writing – review and editing (equal). **Ajoy Kumer:** conceptualization (equal), data curation (equal), formal analysis (equal), methodology (equal), project administration (equal), writing – review and editing (equal).

## Ethics Statement

Approval from the Institutional Animal Ethical Committee of the Department of Pharmaceutical Sciences, Dibrugarh University, Assam, India (institutional regd. No. 1576/GO/a/11/CPCSE dated: 17/2/2012). The current study is approved under Approval No. IAEC/DU/244, dated 17/12/2022. The experiments are performed with the animal models as per the standard norms given by the Institutional Animal Care Committee, CPCSEA, India.

## Conflicts of Interest

The authors declare no conflicts of interest.

## Supporting information


Table S1.

## Data Availability

The data incorporated in the manuscript is self generated after conducting various experiments.No data has been copied from other sources.All the data produced are original.
