# Supplementary material for: Design and Development of Xanthone Hybrid for Potent Anti‐Inflammatory Effects: Synthesis and Evaluation
Source: J Cell Mol Med. 2025 Mar 24;29(6):e70477. doi: 10.1111/jcmm.70477 (PMC11932163; doi:10.1111/jcmm.70477)
Supplement: Supplementary file 1 — Table S1. [file JCMM-29-e70477-s001.docx]

**Table 1: Analysis of the various molecular properties of the designed compounds.( the synthesized compounds)**

| **Compouds**  **Code** | **Molecular weight of compounds** | **Molecular formula of compounds** | **MlogP values** | **nRB** | **TPSA** | **nOH** | **nOH-NH** | **nViolations** | **Volume** |
| --- | --- | --- | --- | --- | --- | --- | --- | --- | --- |
| A1 | 410.43 |  | 4.14 | 8 | 117.95 | 8 | 3 | 0 | 359.05 |
| A2 | 546.62 |  | 5.80 | 15 | 124.79 | 10 | 2 | 2 | 496.64 |
| A3 | 340.29 |  | 2.58 | 3 | 117.95 | 8 | 3 | 0 | 275.04 |
| A4 | 420.38 |  | 3.18 | 6 | 124.79 | 10 | 2 | 0 | 345.43 |
| A5 | 354.32 |  | 2.59 | 4 | 117.95 | 8 | 3 | 0 | 291.84 |
| A6 | 448.44 |  | 3.20 | 8 | 124.79 | 10 | 2 | 0 | 379.03 |
| A7 | 368.35 |  | 2.86 | 5 | 117.95 | 8 | 3 | 0 | 308.64 |
| A8 | 476.49 |  | 3.74 | 10 | 124.79 | 10 | 2 | 0 | 412.63 |
| A9 | 382.37 |  | 3.13 | 6 | 117.95 | 8 | 3 | 0 | 325.44 |
| A10 | 504.54 |  | 4.28 | 12 | 124.79 | 10 | 2 | 1 | 446.24 |
| A11 | 396.40 |  | 3.64 | 7 | 117.95 | 8 | 3 | 0 | 342.25 |
| A12 | 532.60 |  | 5.29 | 14 | 124.79 | 10 | 2 | 2 | 479.84 |
| A13 | 461.51 |  | 5.34 | 8 | 101.16 | 7 | 3 | 1 | 413.43 |
| A14 | 644.77 |  | 8.91 | 15 | 99.00 | 8 | 2 | 2 | 592.94 |
| A15 | 389.36 |  | 4.42 | 3 | 105.06 | 7 | 3 | 0 | 323.19 |
| A16 | 518.52 |  | 6.86 | 6 | 99.00 | 8 | 2 | 2 | 441.72 |
| A17 | 403.39 |  | 4.43 | 4 | 105.06 | 7 | 3 | 0 | 339.99 |
| A18 | 546.58 |  | 6.88 | 8 | 99.00 | 8 | 2 | 2 | 475.33 |
| A19 | 417.42 |  | 4.70 | 5 | 105.06 | 7 | 3 | 0 | 356.79 |
| A20 | 574.63 |  | 7.42 | 10 | 99.00 | 8 | 2 | 2 | 508.93 |
| A21 | 432.44 |  | 4.97 | 6 | 105.06 | 7 | 3 | 0 | 373.59 |
| A22 | 602.69 |  | 7.97 | 12 | 99.00 | 8 | 2 | 2 | 542.53 |
| A23 | 445.47 |  | 5.48 | 7 | 105.06 | 7 | 3 | 1 | 390.39 |
| A24 | 630.74 |  | 8.68 | 14 | 99.00 | 8 | 2 | 2 | 576.14 |
| A25 | 475.45 |  | 4.15 | 7 | 139.21 | 9 | 3 | 0 | 400.94 |
| A26 | 690.71 |  | 6.32 | 14 | 167.29 | 12 | 2 | 3 | 597.24 |
| A27 | 662.65 |  | 5.31 | 12 | 167.29 | 12 | 2 | 3 | 563.63 |
| A28 | 461.43 |  | 3.64 | 6 | 139.21 | 9 | 3 | 0 | 384.14 |
| A29 | 447.40 |  | 3.37 | 5 | 139.21 | 9 | 3 | 0 | 367.34 |
| A30 | 634.60 |  | 4.76 | 10 | 167.29 | 12 | 2 | 2 | 530.03 |
| A31 | 606.54 |  | 4.22 | 8 | 167.29 | 12 | 2 | 2 | 496.43 |
| A32 | 433.37 |  | 3.10 | 4 | 139.21 | 9 | 3 | 0 | 350.54 |
| A33 | 419.35 |  | 3.09 | 3 | 139.21 | 9 | 3 | 0 | 333.74 |
| A34 | 578.49 |  | 4.20 | 6 | 167.29 | 12 | 2 | 2 | 462.82 |
| A35 | 704.73 |  | 6.82 | 15 | 167.29 | 12 | 2 | 3 | 614.04 |
| A36 | 491.50 |  | 4.01 | 8 | 135.30 | 9 | 3 | 0 | 423.98 |
| A37 | 410.43 |  | 3.75 | 8 | 117.95 | 8 | 3 | 0 | 359.05 |
| A38 | 560.65 |  | 5.52 | 16 | 124.79 | 10 | 2 | 2 | 496.62 |
| A39 | 420.38 |  | 2.39 | 6 | 124.79 | 10 | 2 | 0 | 345.43 |
| A40 | 340.29 |  | 2.19 | 3 | 117.95 | 8 | 3 | 0 | 275.04 |
| A41 | 354.32 |  | 2.20 | 4 | 117.95 | 8 | 3 | 0 | 291.84 |
| A42 | 448.44 |  | 2.42 | 8 | 124.79 | 10 | 2 | 0 | 379.03 |
| A43 | 476.49 |  | 2.96 | 10 | 124.79 | 10 | 2 | 0 | 412.63 |
| A44 | 368.35 |  | 2.47 | 5 | 117.95 | 8 | 3 | 0 | 308.64 |
| A45 | 382.37 |  | 2.74 | 6 | 117.95 | 8 | 3 | 0 | 325.44 |
| A46 | 504.54 |  | 3.50 | 12 | 124.79 | 10 | 2 | 1 | 446.24 |
| A47 | 532.60 |  | 4.51 | 14 | 124.79 | 10 | 2 | 1 | 479.84 |
| A48 | 396.40 |  | 3.25 | 7 | 117.95 | 8 | 3 | 0 | 342.25 |
| A49 | 428.49 |  | 3.40 | 8 | 115.39 | 8 | 4 | 0 | 387.98 |
| A50 | 582.74 |  | 4.30 | 15 | 119.67 | 10 | 4 | 1 | 554.51 |
| A51 | 456.50 |  | 1.68 | 6 | 119.67 | 10 | 4 | 0 | 403.29 |
| A52 | 358.35 |  | 1.83 | 3 | 115.39 | 8 | 4 | 0 | 303.97 |
| A53 | 327.38 |  | 1.84 | 4 | 115.39 | 8 | 4 | 0 | 320.77 |
| A54 | 484.55 |  | 1.71 | 8 | 119.67 | 10 | 4 | 0 | 436.89 |
| A55 | 386.40 |  | 2.11 | 5 | 115.39 | 8 | 4 | 0 | 337.57 |
| A56 | 512.61 |  | 2.25 | 10 | 119.67 | 10 | 4 | 1 | 470.50 |
| A57 | 400.43 |  | 2.38 | 6 | 115.39 | 8 | 4 | 0 | 354.38 |
| A58 | 540.66 |  | 2.79 | 12 | 119.67 | 10 | 4 | 1 | 504.10 |
| A59 | 414.46 |  | 2.89 | 7 | 115.39 | 8 | 4 | 0 | 371.18 |
| A60 | 568.72 |  | 3.80 | 14 | 119.67 | 10 | 4 | 1 | 537.71 |
| A61 | 341.28 |  | 1.65 | 3 | 130.85 | 9 | 3 | 0 | 270.88 |
| A62 | 422.36 |  | 1.32 | 6 | 150.57 | 12 | 2 | 1 | 337.11 |
| A63 | 355.31 |  | 1.66 | 4 | 130.85 | 9 | 3 | 0 | 287.68 |
| A64 | 450.41 |  | 1.35 | 8 | 150.57 | 12 | 2 | 1 | 370.72 |
| A65 | 369.33 |  | 1.94 | 5 | 130.85 | 9 | 3 | 0 | 304.49 |
| A66 | 478.46 |  | 1.89 | 10 | 150.57 | 12 | 2 | 1 | 404.32 |
| A67 | 383.36 |  | 2.21 | 6 | 130.85 | 9 | 3 | 0 | 321.29 |
| A68 | 506.52 |  | 2.43 | 12 | 150.57 | 12 | 2 | 2 | 437.92 |
| A69 | 397.39 |  | 2.71 | 7 | 130.85 | 9 | 3 | 0 | 338.09 |
| A70 | 534.57 |  | 3.44 | 14 | 150.57 | 12 | 2 | 2 | 471.53 |
| A71 | 411.41 |  | 3.22 | 8 | 130.85 | 9 | 3 | 0 | 354.89 |
| A72 | 562.63 |  | 4.45 | 16 | 150.57 | 12 | 2 | 2 | 505.13 |
| B1 | 394.43 |  | 4.67 | 8 | 97.73 | 7 | 2 | 0 | 351.03 |
| B2 | 324.29 |  | 3.11 | 3 | 97.73 | 7 | 2 | 0 | 267.02 |
| B3 | 338.32 |  | 3.12 | 4 | 97.73 | 7 | 2 | 0 | 283.82 |
| B4 | 352.35 |  | 3.39 | 5 | 97.73 | 7 | 2 | 0 | 300.62 |
| B5 | 366.37 |  | 3.66 | 6 | 97.73 | 7 | 2 | 0 | 317.43 |
| B6 | 380.40 |  | 4.17 | 7 | 97.73 | 7 | 2 | 0 | 334.23 |
| B7 | 443.50 |  | 6.51 | 8 | 84.83 | 6 | 2 | 1 | 399.18 |
| B8 | 373.36 |  | 4.95 | 3 | 84.83 | 6 | 2 | 0 | 315.17 |
| B9 | 387.39 |  | 4.96 | 4 | 84.83 | 6 | 2 | 0 | 331.97 |
| B10 | 401.42 |  | 5.32 | 5 | 84.83 | 6 | 2 | 1 | 348.77 |
| B11 | 415.44 |  | 5.50 | 6 | 84.83 | 6 | 2 | 1 | 365.57 |
| B12 | 429.47 |  | 6.01 | 7 | 84.83 | 6 | 2 | 1 | 382.38 |
| B13 | 473.48 |  | 5.18 | 8 | 118.98 | 8 | 2 | 1 | 409.73 |
| B14 | 403.35 |  | 3.62 | 3 | 118.98 | 8 | 2 | 0 | 325.72 |
| B15 | 417.37 |  | 3.63 | 4 | 118.98 | 8 | 2 | 0 | 342.52 |
| B16 | 431.40 |  | 3.9 | 5 | 118.98 | 8 | 2 | 0 | 359.32 |
| B17 | 445.43 |  | 4.17 | 6 | 118.98 | 8 | 2 | 0 | 376.12 |
| B18 | 459.45 |  | 4.68 | 7 | 118.98 | 8 | 2 | 0 | 392.93 |
| B19 | 394.43 |  | 4.28 | 8 | 97.73 | 7 | 2 | 0 | 351.03 |
| B20 | 324.29 |  | 2.71 | 3 | 97.73 | 7 | 2 | 0 | 267.02 |
| B21 | 352.35 |  | 3.00 | 5 | 97.73 | 7 | 2 | 0 | 300.62 |
| B22 | 366.37 |  | 3.27 | 6 | 97.73 | 7 | 2 | 0 | 317.43 |
| B23 | 380.40 |  | 3.77 | 2 | 97.73 | 7 | 2 | 0 | 334.23 |
| B24 | 412.49 |  | 3.92 | 8 | 95.17 | 7 | 3 | 0 | 379.96 |
| B25 | 342.35 |  | 2.36 | 3 | 95.17 | 7 | 3 | 0 | 295.95 |
| B26 | 356.38 |  | 2.37 | 4 | 95.17 | 7 | 3 | 0 | 312.75 |
| B27 | 384.43 |  | 2.91 | 6 | 95.17 | 7 | 3 | 0 | 346.36 |
| B28 | 370.40 |  | 2.64 | 5 | 95.17 | 7 | 3 | 0 | 329.56 |
| B29 | 398.46 |  | 3.42 | 7 | 95.17 | 7 | 3 | 0 | 363.16 |
| C1 | 410.43 |  | 4.17 | 8 | 117.95 | 8 | 3 | 0 | 359.05 |
| C2 | 340.29 |  | 2.61 | 3 | 117.95 | 8 | 3 | 0 | 275.04 |
| C3 | 354.32 |  | 2.62 | 4 | 117.95 | 8 | 3 | 0 | 291.84 |
| C4 | 382.37 |  | 3.16 | 6 | 117.95 | 8 | 3 | 0 | 325.44 |
| C5 | 396.40 |  | 3.67 | 7 | 117.95 | 8 | 3 | 0 | 342,25 |
| C6 | 445.47 |  | 5.51 | 7 | 105.06 | 7 | 3 | 1 | 390.39 |
| C7 | 431.44 |  | 5.00 | 6 | 105.06 | 7 | 3 | 1 | 373.59 |
| C8 | 417 |  | 4.73 | 5 | 105.06 | 7 | 3 | 0 | 356.79 |
| C9 | 403.39 |  | 4.46 | 4 | 105.06 | 7 | 3 | 0 | 339.99 |
| C10 | 389.36 |  | 4.45 | 3 | 105.06 | 7 | 3 | 0 | 323.19 |
| C11 | 459.50 |  | 6.01 | 8 | 105.06 | 7 | 3 | 1 | 407.19 |
| C12 | 489.48 |  | 4.68 | 8 | 139.21 | 9 | 3 | 0 | 417.75 |
| C13 | 419.35 |  | 3.12 | 3 | 139.21 | 9 | 3 | 0 | 333.74 |
| C14 | 433.37 |  | 3.13 | 4 | 139.21 | 9 | 3 | 0 | 350.54 |
| C15 | 447.40 |  | 3.40 | 5 | 139.21 | 9 | 3 | 0 | 367.34 |
| C16 | 475.45 |  | 4.18 | 7 | 139.21 | 9 | 3 | 0 | 400.94 |
| C17 | 340.29 |  | 2.22 | 3 | 117.95 | 8 | 3 | 0 | 275.04 |
| C18 | 354.32 |  | 2.23 | 4 | 117.95 | 8 | 3 | 0 | 291.84 |
| C19 | 368.35 |  | 2.50 | 5 | 117.95 | 8 | 3 | 0 | 308.64 |
| C20 | 382.37 |  | 2.77 | 6 | 117.95 | 8 | 3 | 0 | 325.44 |
| C21 | 396.40 |  | 3.28 | 7 | 117.95 | 8 | 3 | 0 | 342.25 |
| C22 | 428.49 |  | 3.43 | 8 | 115.39 | 8 | 4 | 0 | 387.98 |
| C23 | 358.35 |  | 1.86 | 3 | 115.39 | 8 | 4 | 0 | 303.97 |
| C24 | 372.38 |  | 1.87 | 4 | 115.39 | 8 | 4 | 0 | 320.77 |
| C25 | 386.40 |  | 2.15 | 5 | 115.39 | 8 | 4 | 0 | 337.57 |
| C26 | 400.43 |  | 2.42 | 6 | 115.39 | 8 | 4 | 0 | 354.38 |
| C27 | 414.46 |  | 2.92 | 7 | 115.39 | 8 | 4 | 0 | 371.18 |

(nRB = number of rotatable bond, TPSA=Total Polar Surface Area, nOH&NH= number of OH & NH bonds)

Table 2: Toxicity data analysis of the designed compound by ORISIS DATA WARRIOR

| **Compound no.** | **Mutagenic** | **Tumorigenic** | **Irritant** | **Reproductive effect** |
| --- | --- | --- | --- | --- |
| A1 | High | None | Low | None |
| A3 | High | None | Low | None |
| A4 | High | None | High | None |
| A5 | High | None | Low | None |
| A6 | High | None | High | None |
| A7 | High | None | Low | None |
| A8 | High | None | High | None |
| A9 | High | None | Low | None |
| A10 | High | None | High | None |
| A11 | High | None | Low | None |
| A13 | High | None | Low | None |
| A15 | High | None | Low | None |
| A16 | High | None | High | None |
| A17 | High | None | Low | None |
| A19 | High | None | Low | None |
| A21 | High | None | Low | None |
| A23 | High | None | Low | None |
| A25 | High | None | Low | None |
| A28 | High | None | Low | None |
| A29 | High | None | Low | None |
| A32 | High | None | Low | None |
| A33 | High | High | Low | High |
| A36 | High | None | Low | None |
| A37 | High | None | Low | None |
| A39 | High | None | High | None |
| A40 | High | None | Low | None |
| A41 | High | None | Low | None |
| A42 | High | None | High | None |
| A43 | High | None | High | None |
| A44 | High | None | Low | None |
| A45 | High | None | Low | None |
| A46 | High | None | High | None |
| A47 | High | None | High | None |
| A48 | High | None | Low | None |
| A49 | High | None | Low | Low |
| A50 | High | None | High | Low |
| A51 | High | None | High | None |
| A52 | High | None | Low | None |
| A53 | High | None | Low | Low |
| A54 | High | None | High | Low |
| A55 | High | None | Low | Low |
| A56 | High | None | High | Low |
| A57 | High | None | Low | Low |
| A58 | High | None | High | Low |
| A59 | High | None | Low | Low |
| A60 | High | None | High | Low |
| A61 | High | None | Low | None |
| A62 | High | None | High | None |
| A63 | High | None | Low | None |
| A64 | High | None | High | None |
| A65 | High | None | Low | None |
| A66 | High | None | High | None |
| A67 | High | None | Low | None |
| A69 | High | None | Low | None |
| A71 | High | None | Low | None |
| B1 | High | None | Low | None |
| B2 | High | None | Low | None |
| B3 | High | None | Low | None |
| B4 | High | None | Low | None |
| B5 | High | None | Low | None |
| B6 | High | None | Low | None |
| B7 | High | None | Low | None |
| B8 | High | None | Low | None |
| B9 | High | None | Low | None |
| B10 | High | None | Low | None |
| B11 | High | None | Low | None |
| B12 | High | None | Low | None |
| B13 | High | None | Low | None |
| B14 | High | High | Low | High |
| B15 | High | None | Low | None |
| B16 | High | None | Low | None |
| B17 | High | None | Low | None |
| B18 | High | None | Low | None |
| B19 | High | None | Low | None |
| B20 | High | None | Low | None |
| B21 | High | None | Low | None |
| B22 | High | None | Low | None |
| B23 | High | None | Low | None |
| B24 | High | None | Low | None |
| B25 | High | None | Low | Low |
| B26 | High | None | Low | Low |
| B27 | High | None | Low | Low |
| B28 | High | None | Low | Low |
| B29 | High | None | Low | Low |
| C1 | High | None | Low | None |
| C2 | High | None | Low | None |
| C3 | High | None | Low | None |
| C4 | High | None | Low | None |
| C5 | High | None | Low | None |
| C6 | High | None | Low | None |
| C7 | High | None | Low | None |
| C8 | High | None | Low | None |
| C9 | High | None | Low | None |
| C10 | High | None | Low | None |
| C11 | High | None | Low | None |
| C12 | High | None | Low | None |
| C13 | High | None | Low | None |
| C14 | High | High | Low | High |
| C15 | High | None | Low | None |
| C16 | High | None | Low | None |
| C17 | High | None | Low | None |
| C18 | High | None | Low | None |
| C19 | High | None | Low | None |
| C20 | High | None | Low | None |
| C21 | High | None | Low | None |
| C22 | High | None | Low | None |
| C23 | High | None | Low | Low |
| C24 | High | None | Low | None |
| C25 | High | None | Low | Low |
| C26 | High | None | Low | Low |
| C27 | High | None | Low | Low |

Table 3: Navigation the ADME properties of the designed compounds by SWISS ADME.

| **Compound no.** | **Log P** | **Solubility** | **Log kp**  **(skin permeation)** | **Bioavailability score** | **Synthetic Accessibility** | **GI absorption** | **BBB permeant** |
| --- | --- | --- | --- | --- | --- | --- | --- |
| A1 | 3.59 | -5.96 | -6.11 | 0.55 | 3.39 | High | No |
| A3 | 2.29 | -4.58 | -6.63 | 0.55 | 3.11 | High | No |
| A4 | 2.9 | -5.1 | -6.86 | 0.55 | 3.47 | High | No |
| A5 | 2.17 | -4.43 | -6.43 | 0.55 | 3.04 | High | No |
| A6 | 3.39 | -4.9 | -7.17 | 0.55 | 3.55 | High | No |
| A7 | 2.17 | -4.85 | -6.62 | 0.55 | 3.12 | High | No |
| A8 | 3.71 | -5.64 | -6.84 | 0.55 | 3.72 | High | No |
| A9 | 3.01 | -5.21 | -6.45 | 0.55 | 3.21 | High | No |
| A10 | 3.96 | -6.38 | -6.5 | 0.55 | 3.94 | Low | No |
| A11 | 3.26 | -5.59 | -6.28 | 0.55 | 3.29 | High | No |
| A13 | 4.26 | -7.53 | -5.16 | 0.55 | 3.55 | Low | No |
| A15 | 3.13 | -6.14 | -5.68 | 0.55 | 3.15 | High | No |
| A16 | 4.56 | -8.61 | -4.61 | 0.55 | 4.57 | Low | No |
| A17 | 3.49 | -6.41 | -5.67 | 0.55 | 3.23 | High | No |
| A19 | 3.71 | -6.78 | -5.5 | 0.55 | 3.33 | High | No |
| A21 | 3.9 | -7.15 | -5.33 | 0.55 | 3.43 | High | No |
| A23 | 3.39 | -6.76 | -6.25 | 0.55 | 3.38 | Low | No |
| A25 | 3.1 | -6.39 | -6.41 | 0.55 | 3.29 | Low | No |
| A28 | 2.89 | -6.02 | -6.58 | 0.55 | 3.18 | Low | No |
| A29 | 2.89 | -6.02 | -6.58 | 0.55 | 3.18 | Low | No |
| A32 | 2.66 | -5.65 | -6.75 | 0.55 | 3.12 | High | No |
| A33 | 1.92 | -5.25 | -6.55 | 0.55 | 3.26 | High | No |
| A36 | 3.65 | -7.13 | -6.08 | 0.55 | 3.5 | Low | No |
| A37 | 3.47 | -5.73 | -6.27 | 0.55 | 3.36 | High | No |
| A39 | 3.08 | -5.19 | -6.63 | 0.55 | 3.58 | High | No |
| A40 | 2.72 | -4.64 | -7.17 | 0.55 | 3.38 | High | No |
| A41 | 3.72 | -5.7 | -6.05 | 0.55 | 3.28 | High | No |
| A42 | 3.97 | -5.6 | -6.2 | 0.55 | 3.33 | High | No |
| A43 | 4.78 | -5.79 | -6.74 | 0.55 | 3.83 | High | No |
| A44 | 4.94 | -6.16 | -6.58 | 0.55 | 3.92 | Low | No |
| A45 | 4.15 | -5.97 | -6.03 | 0.55 | 3.34 | High | No |
| A46 | 4.43 | -6.33 | -5.78 | 0.55 | 3.5 | High | No |
| A47 | 5.19 | -7.28 | -6.07 | 0.55 | 4.19 | Low | No |
| A48 | 5.19 | -7.28 | -5.7 | 0.55 | 4.19 | Low | No |
| A49 | 4.74 | -6.72 | -6.1 | 0.55 | 3.56 | High | No |
| A50 | 5.43 | -6.68 | -7.14 | 0.55 | 3.92 | High | No |
| A51 | 7.8 | -7.95 | -6.4 | 0.55 | 5.24 | High | No |
| A52 | 5.44 | -5.18 | -6.55 | 0.55 | 4.39 | High | No |
| A53 | 4.26 | -5.3 | -8.18 | 0.55 | 3.73 | High | No |
| A54 | 4.55 | -5.19 | -7.12 | 0.55 | 3.64 | High | No |
| A55 | 4.38 | -3.63 | -7.85 | 0.55 | 4.1 | High | No |
| A56 | 3.24 | -4.22 | -6.21 | 0.55 | 3.31 | High | No |
| A57 | 4.68 | -4.37 | -6.77 | 0.55 | 4.21 | High | No |
| A58 | 4.91 | -5.94 | -6.05 | 0.55 | 3.37 | High | No |
| A59 | 6.87 | -4.95 | -6.44 | 0.55 | 4.78 | High | No |
| A60 | 5.06 | -4.95 | -5.77 | 0.55 | 3.81 | High | No |
| A61 | 2.16 | -4.61 | -6.8 | 0.55 | 3.14 | High | No |
| A62 | 2.28 | -5.16 | -7.2 | 0.55 | 3.46 | Low | No |
| A63 | 2.09 | -4.51 | -6.96 | 0.55 | 3.13 | High | No |
| A64 | 2.68 | -4.96 | -7.51 | 0.55 | 3.56 | Low | No |
| A65 | 2.5 | -4.88 | -6.79 | 0.55 | 3.12 | High | No |
| A66 | 3.21 | -5.7 | -7.17 | 0.55 | 3.68 | Low | No |
| A67 | 2.78 | -5.26 | -6.61 | 0.55 | 3.21 | High | No |
| A69 | 2.99 | -5.62 | -6.45 | 0.55 | 3.28 | High | No |
| A71 | 3.54 | -6.55 | -5.98 | 0.55 | 3.51 | Low | No |
| B1 | 3.81 | -5.94 | -6.28 | 0.55 | 4.99 | High | No |
| B2 | 2.76 | -6.46 | -6.43 | 0.55 | 3.36 | High | No |
| B3 | 2.17 | -6.3 | -5.53 | 0.55 | 3.07 | High | No |
| B4 | 4.63 | -7.2 | -5.36 | 0.55 | 3.04 | High | No |
| B5 | 4.86 | -5.9 | -5.19 | 0.55 | 3.38 | High | No |
| B6 | 5.17 | -4.52 | -4.07 | 0.55 | 3.47 | High | No |
| B7 | 6.07 | -4.43 | -4.59 | 0.55 | 3.55 | High | No |
| B8 | 4.79 | -6.14 | -4.74 | 0.55 | 3.82 | Low | No |
| B9 | 4.72 | -6.51 | -4.57 | 0.55 | 3.38 | High | No |
| B10 | 5.19 | -6.89 | -4.4 | 0.55 | 3.44 | High | No |
| B11 | 5.52 | -8.81 | -4.24 | 0.55 | 3.49 | High | No |
| B12 | 5.72 | -7.43 | -4.98 | 0.55 | 3.59 | High | No |
| B13 | 5.36 | -7.33 | -5.51 | 0.55 | 3.7 | Low | No |
| B14 | 4.34 | -7.7 | -5.66 | 0.55 | 3.77 | Low | No |
| B15 | 4.37 | -8.08 | -5.15 | 0.55 | 3.5 | High | No |
| B16 | 4.37 | -8.44 | -5.15 | 0.55 | 3.38 | High | No |
| B17 | 4.5 | -8.43 | -5.15 | 0.55 | 3.38 | High | No |
| B18 | 4.5 | -7.04 | -5.7 | 0.55 | 3.65 | High | No |
| B19 | 4.5 | -6.94 | -5.85 | 0.55 | 3.65 | High | No |
| B20 | 4.16 | -6.94 | -5.69 | 0.55 | 3.65 | High | No |
| B21 | 4.3 | -8.05 | -5.52 | 0.55 | 3.24 | High | No |
| B22 | 4.51 | -8.05 | -5.35 | 0.55 | 3.3 | High | No |
| B23 | 4.79 | -8.05 | -5.53 | 0.55 | 3.3 | High | No |
| B24 | 4.97 | -5.64 | -6.05 | 0.55 | 3.41 | High | No |
| B25 | 5.64 | -5.55 | -6.94 | 0.55 | 3.52 | High | No |
| B26 | 4.61 | -5.91 | -6.61 | 0.55 | 3.88 | High | No |
| B27 | 3.04 | -6.28 | -6.78 | 0.55 | 3.7 | High | No |
| B28 | 3.86 | -6.66 | -6.44 | 0.55 | 3.3 | High | No |
| B29 | 3.01 | -6.61 | -6.11 | 0.55 | 3.39 | High | No |
| C1 | 4.1 | -5.23 | -6.63 | 0.55 | 3.28 | High | No |
| C2 | 3.44 | -3.79 | -6.79 | 0.55 | 3.46 | High | No |
| C3 | 2.13 | -4.53 | -6.62 | 0.55 | 3.48 | High | No |
| C4 | 2,26 | -4.15 | -6.45 | 0.55 | 3.18 | High | No |
| C5 | 2.77 | -4.9 | -6.28 | 0.55 | 3.16 | High | No |
| C6 | 2.89 | -5.96 | -5.33 | 0.55 | 3.21 | High | No |
| C7 | 3.21 | -4.58 | -5.5 | 0.55 | 3.3 | High | No |
| C8 | 3.36 | -4.48 | -5.67 | 0.55 | 3.38 | High | No |
| C9 | 3.59 | -4.85 | -5.83 | 0.55 | 3.53 | High | No |
| C10 | 3.27 | -5.21 | -5.68 | 0.55 | 3.42 | High | No |
| C11 | 3.14 | -5.59 | -5.16 | 0.55 | 3.33 | High | No |
| C12 | 2.88 | -7.15 | -6.08 | 0.55 | 3.27 | High | No |
| C13 | 4.12 | -6.78 | -6.59 | 0.55 | 3.24 | High | No |
| C14 | 3.29 | -6.41 | -6.58 | 0.55 | 3.64 | Low | No |
| C15 | 2.28 | -6.04 | -6.25 | 0.55 | 3.56 | Low | No |
| C16 | 2.27 | -6.14 | -6.79 | 0.55 | 3.3 | High | No |
| C17 | 3.12 | -7.53 | -6.94 | 0.55 | 3.25 | Low | No |
| C18 | 2.21 | -7.13 | -6.77 | 0.55 | 3.44 | Low | No |
| C19 | 2.37 | -5.75 | -6.61 | 0.55 | 3.1 | High | No |
| C20 | 2.58 | -6.02 | -6.44 | 0.55 | 3.12 | High | No |
| C21 | 2.85 | -6.76 | -6,62 | 0.55 | 3.14 | High | No |
| C22 | 3.19 | -4.35 | -7.14 | 0.55 | 3.24 | High | No |
| C23 | 3.69 | -4.25 | -7.29 | 0.55 | 3.35 | High | No |
| C24 | 2.72 | -4.62 | -7.12 | 0.55 | 3.71 | High | No |
| C25 | 2.17 | -4.99 | -6.95 | 0.55 | 3.52 | High | No |
| C26 | 2.87 | -5.33 | -6.79 | 0.55 | 3.43 | High | No |
| C27 | 2.87 | -3.95 | -6.79 | 0.55 | 3.41 | High | No |
